# High‐Throughput Mechanical Characterization of Single Microgel Particles by Fluidic Force Microscopy

**DOI:** 10.1002/smll.202505367

**Published:** 2025-08-08

**Authors:** Agnes Specht, Steffen Trippmacher, Nadine Raßmann, Tamino Rößler, Katinka Theis, Krystyna Albrecht, Nicolas Helfricht, Jürgen Groll, Georg Papastavrou

**Affiliations:** ^1^ Department of Physical Chemistry II University of Bayreuth Universitätsstraße 30 95447 Bayreuth Germany; ^2^ Department for Functional Medicine and Dentistry University of Würzburg Pleicherwall 2 97070 Würzburg Germany; ^3^ Bavarian Polymer Institute University of Bayreuth Universitätsstraße 30 95447 Bayreuth Germany; ^4^ Bayreuth Center of Colloids and Interfaces University of Bayreuth Universitätsstraße 30 95447 Bayreuth Germany

**Keywords:** atomic force microscopy, fluidic force microscopy, mechanics of soft matter, microgels, nanoindentation

## Abstract

The mechanical characterization of soft particulate materials by nanoindentation based on atomic force microscopy (AFM) is a well‐established technique in materials science. However, this technique is very time‐consuming for micrometer‐sized particles as the indenter has to be centered on the particle apex. As microgels have a broad distribution of Young's moduli, it is important to measure many particles to achieve statistically reliable data. Here, a new approach to nanoindentation is presented where the roles of the sample and the indenter are reversed. The technique is based on fluidic force microscopy (FluidFM): The microgel particle is aspirated to the aperture of a cantilever with an internal channel connected to a microfluidic controller, and the microgel particle is subsequently ramped onto a flat substrate. The experimental validation is carried out with two different types of microgels: ene‐functionalized polyoxazoline crosslinked with thiol‐functionalized hyaluronic acid (POx‐HASH) and polyacrylamide (PAAm). It is demonstrated that in combination with the simplified double contact model, the “inverted” nanoindentation can determine Young's moduli of microgel particles about 5–10 times faster. Moreover, the here‐presented technique allows for performing indentation measurements on different substrates. Thereby, it becomes possible to elucidate the role of adhesion in the nanoindentation process.

## Graphical abstract

The combination of atomic force microscopy and nanofluidics (FluidFM) allows for a new approach for the mechanical characterization of particulate soft matter in the micrometer regime by inverted nanoindentation. Based on the simplified double contact model, the Young's moduli of microgel particles can be determined much faster than by classical nanoindentation, allowing for statistically relevant data sets.

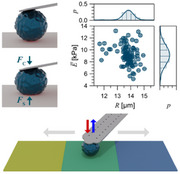

## 1 Introduction

Microgels have received much attention in recent years as they found applications in biocatalysis, sensing, and drug delivery.^[^ ^1^, ^2^ ^]^ For some other applications, it is their mechanical versatility that makes them of primary interest:^[^ ^3^, ^4^ ^]^ In particular, microgel particles are serving as building blocks for granular hydrogels.^[^ ^5^, ^6^ ^]^ The overall properties of a granular hydrogel can be tuned by varying the composition, adhesion, and ratio of different hydrogel microparticles.^[^ ^6^, ^7^, ^8^ ^]^ Granular hydrogels have been increasingly applied in the field of biomaterials, for example, as bioinks for the formation of 3D structures by bioprinting.^[^ ^9^, ^10^, ^11^, ^12^ ^]^ Besides their use in additive biofabrication, microgels can also be injected to restore tissue functionality^[^ ^13^, ^14^ ^]^ and transmit electrical signals.^[^ ^15^, ^16^ ^]^ Since microgels can be fabricated with similar dimensions and elastic moduli as cells,^[^ ^17^, ^18^ ^]^ the utilization of hybrid materials made from cells and microgel particles is of special interest.^[^ ^19^, ^20^ ^]^

One of the techniques frequently used for determining the Young's modulus of soft materials is nanoindentation by atomic force microscopy (AFM).^[^ ^21^, ^22^, ^23^, ^24^, ^25^ ^]^ For the nanoindentation of thin, soft films, consensus about best practice techniques has formed in the last years, including data evaluation procedures.^[^ ^23^, ^26^, ^27^, ^28^ ^]^ By contrast, the nanoindentation of individual soft particles has been studied less.^[^ ^18^, ^29^, ^30^, ^31^, ^32^, ^33^ ^]^ However, colloidal force spectroscopy by fluidic force microscopy (FluidFM) has also been used for this purpose.^[^ ^34^ ^]^ Recently, it has been demonstrated that the indenter geometry and the underlying continuum model for contact mechanics for fitting the force versus indentation curves have a great influence on the derived Young's moduli when adhesive contributions were taken into account.^[^ ^35^ ^]^ In the case of microparticles, the “classical” Hertz model will underestimate the Young's modulus, as the deformation of the microgel particle at the particle‐substrate contact is not accounted for.^[^ ^35^, ^36^ ^]^ Hence, a so‐called simplified double contact model should be utilized,^[^ ^36^, ^37^ ^]^ which does lead to more consistent results for the elastic properties.^[^ ^30^, ^35^ ^]^ However, there are still several open questions, such as the role of adhesion in the double contact model.^[^ ^35^ ^]^

One major disadvantage of “classical” nanoindentation on single particles is that the indenter must be placed accurately on the particle apex.^[^ ^35^ ^]^ While coarse alignment can be simply achieved by optical microscopy, fine alignment with sub‐µm precision requires so‐called force volume plots.^[^ ^38^, ^39^ ^]^ This fine alignment takes much more time than the acquisition of the actual indentation curves. Hence, it is commonly very time‐consuming to probe particle mechanics by nanoindentation, especially for spherical or conical indenters. In consequence, the acquisition of a statistically relevant number of Young's moduli for individual particles typically takes significant time. However, statistically reliable sampling is important not only for adhesion measurements^[^ ^40^ ^]^ but also for nanoindentation: Large statistical variation of Young's moduli is typically found within one batch of hydrogel particles,^[^ ^17^, ^41^ ^]^ even when prepared by microfluidic methods.^[^ ^30^, ^42^, ^43^ ^]^ Hence, rather than sampling only 5 to 10 particles, a ten times larger data set would most certainly be required to determine statistically significant data sets. Similar experimental challenges have been encountered in determining the elastic properties of living cells by AFM‐based nanoindentation.^[^ ^37^, ^44^, ^45^, ^46^ ^]^ In the case of cells, micelles, and capsules, various other experimental approaches have been suggested to probe mechanical properties,^[^ ^25^, ^47^, ^48^, ^49^ ^]^ such as aspiration by micropipettes^[^ ^50^, ^51^ ^]^ or deformation in microfluidic channels.^[^ ^30^ ^]^ However, only the latter method can be sufficiently automated to allow for the acquisition of very large datasets, i.e., datasets comprising indentation curves of more than 50 particles, when it is combined with automatic image evaluation to quantify the mechanical deformation of the cells or microgels.^[^ ^52^, ^53^ ^]^

Here, we present a novel approach to determine the Young's modulus of microgel particles by nanoindentation using AFM in combination with nanofluidics. “Classical” nanoindentation can determine the elastic properties on small length scales and with indentation depths of tenths of nanometers.^[^ ^54^, ^55^ ^]^ Our approach is based on a special atomic force microscopy technique, which is called fluidic force microscopy, or shortly, FluidFM.^[^ ^56^, ^57^, ^58^ ^]^ For FluidFM, a modified AFM cantilever is equipped with an internal micro‐channel that ends in an aperture at the free end of the cantilever. The pressure within the channel is regulated externally by a pressure controller as used for microfluidics. This technique allows for the aspiration of single cells,^[^ ^56^, ^59^ ^]^ bacteria,^[^ ^60^ ^]^ or colloidal particles.^[^ ^40^, ^61^, ^62^, ^63^, ^64^ ^]^ Thereby, adhesive properties or interaction potentials can be determined.^[^ ^40^, ^62^, ^63^, ^64^ ^]^ The microfluidic control over the cantilever's micro‐channel enables the temporary aspiration and allows for a fast and reliable exchange of the probe object. This possibility to exchange particles is a great advantage of the FluidFM technique in comparison to the classical colloidal probe technique, where a colloidal particle is irreversibly immobilized at the cantilever.

The fundamental concept of this study is to develop an “inverted” nanoindentation, where the deformable probe object is ramped versus a fixed flat sample. Here, we utilize the fact that the compression of the microgel particles under investigation takes place simultaneously at two contact points in “classical” nanoindentation: The first at the contact with the AFM‐based indenter and the second with the solid substrate to which the particle is immobilized.^[^ ^35^, ^36^ ^]^ From the viewpoint of continuum contact mechanics, the roles of the indenter and the substrate are exchangeable and can be simply inverted. The resulting “inverted” indentation geometry with an aspirated microgel particle is comparable to the one for wedged or tilted tipless cantilevers in classical nanoindentation. However, by aspirating a single microgel particle to the aperture of a micro‐channeled cantilever, the necessity to align the indenter on the apex of the particle is omitted. The inverted indenter geometry implemented by FluidFM allows for determining the Young's modulus of single particles significantly, by our estimation about 5–10 times faster. Thereby, the natural fluctuation of the elastic moduli can be accounted for much better. The results have been compared to “classical” nanoindentation measurements with a spherical indenter geometry. Moreover, the effect of adhesion on the determination of the mechanical properties can be quantified by “inverted” nanoindentation more conveniently, as the cantilever with the aspirated particle can subsequently be placed on different substrates where nanoindentation experiments are carried out. Moreover, we also studied the influence of the pressure applied to the micro‐channeled cantilever.

## 2 Results and Discussion

### 2.1 Manipulating Microgel Particles by Fluidic Force Microscopy

**Figure 1:**
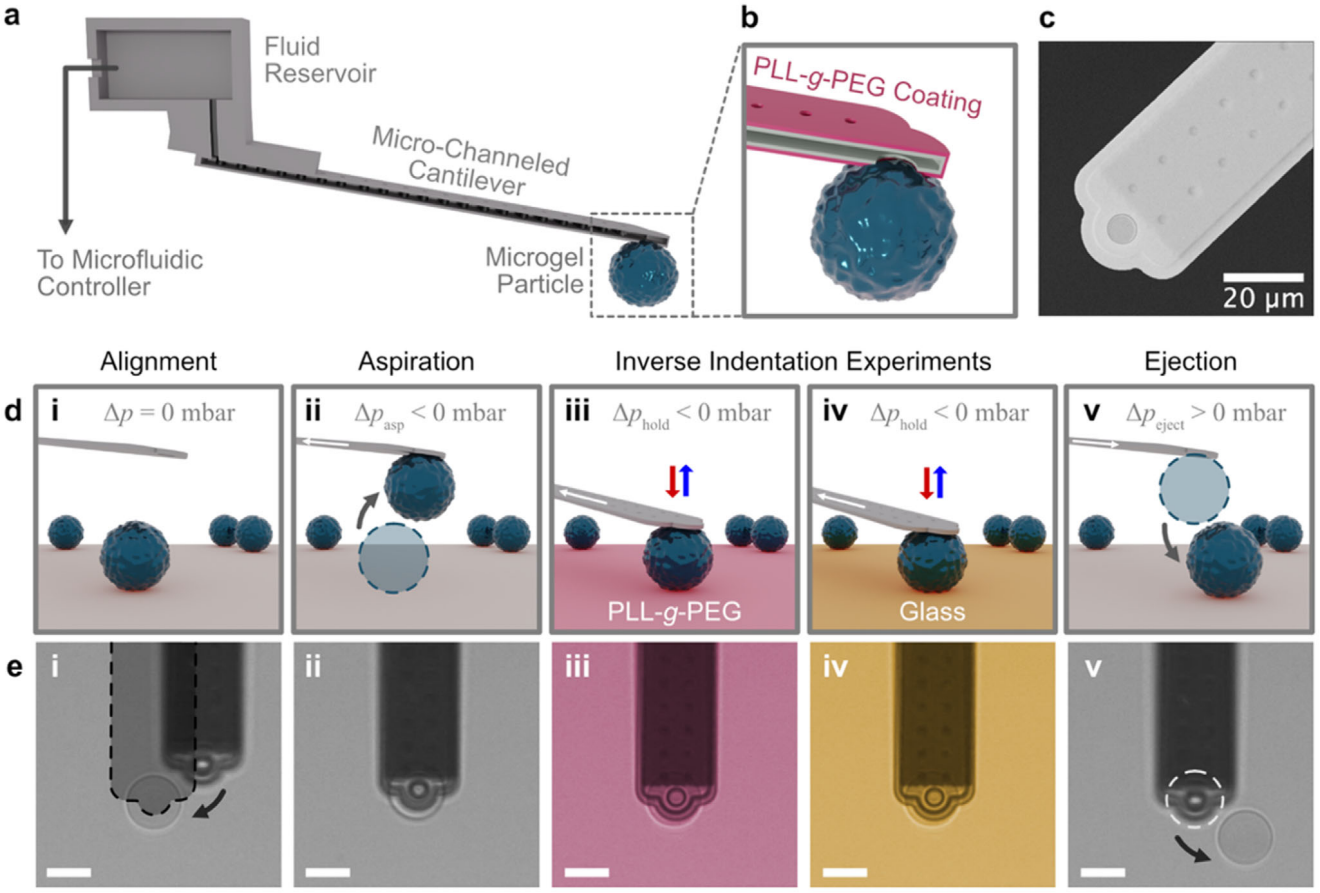
Manipulation of microgel particles by fluidic force microscopy (FluidFM). a) Schematic illustration of a micro‐channeled cantilever, where the internal channel is connected to a microfluidic reservoir. Pressures can be applied to the microfluidic system via an external microfluidic controller. b) Schematic representation of the cross‐sectioned, PLL‐*g*‐PEG‐functionalized cantilever with a microgel particle aspirated to the aperture at the free end of the cantilever. c) SEM image of a micro‐channeled cantilever with a nominal aperture diameter of 8 µm as used in this study. d) Schematic representation and e) optical microscopy images of the sequence for reversibly immobilizing microgel particles and the inverted nanoindentation with those microgel particles (here exemplarily shown for a PAAm particle), including the visual alignment of the cantilever i), the aspiration of a microgel particle ii), indentation experiments on various surfaces iii), iv), and the ejection of the particle v). The white arrows indicate the pressure‐induced fluid flow and the grey arrows the movement of the cantilever and particle, respectively. The scale bars in the optical microscopy images correspond to 20 µm in length.

**Figure** **1**a–c illustrates the most important component of fluidic force microscopy (FluidFM):^[^ ^56^ ^]^ A cantilever with an internal micro‐channel that ends in a defined aperture at the free end of the cantilever and that is connected via a reservoir to a microfluidic controller (cf. Figure 1a). Figure 1c shows a scanning electron microscopy (SEM) image of a micro‐channeled cantilever, which we have used for the experiments in this study. These cantilevers had a nominal aperture of 8 µm in diameter. Before the experiment, the cantilever was additionally coated with PLL‐*g*‐PEG to suppress the adhesion to the aspirated microgel beads (cf. Figure 1b). The FluidFM technique allows not only to aspirate cells^[^ ^56^, ^57^, ^58^ ^]^ but also to manipulate colloidal microparticles that are temporarily immobilized at the cantilever's aperture by applying an underpressure (cf. Figure 1b).^[^ ^61^, ^62^, ^64^ ^]^

Figure 1d shows the particle manipulation sequence for microgel particles in a schematic representation:^[^ ^40^, ^62^, ^64^ ^]^ A corresponding and representative experimental manipulation sequence, as followed by optical light microscopy, is shown in Figure 1e. i) In the beginning, the cantilever is optically aligned above a single microgel particle. The radii of the microgel particles used here ranged between 12 and 16 µm. An aspiration pressure of *p*_asp_ = −800 mbar was applied using the microfluidic controller. This underpressure induces a fluid flow towards the aperture of the micro‐channeled cantilever. In consequence, a single microgel particle is aspirated to the aperture and is temporarily immobilized ii). During the immobilization, the magnitude of the underpressure has been reduced to *p*_hold_ = −100 mbar. We decided on aperture sizes of 8 µm in diameter in order to provide the best stabilization for the immobilized particles. iii) With the aspirated particle, so‐called force versus distance curves can be acquired, analogously to the ones performed with solid particles as colloidal probes.^[^ ^40^, ^61^, ^62^, ^64^ ^]^ As previously demonstrated, not only solid colloids but also soft core‐shell and hydrogel particles can be immobilized at the aperture of a micro‐channeled cantilever.^[^ ^65^ ^]^ Due to the immobilization of the particle on the cantilever, the lateral position of the sample can be varied easily. Thus, the FluidFM technique also allows for easily probing substrates with different chemical functionalities. Here, the interaction with two different substrates has been studied, namely PLL‐*g*‐PEG‐modified glass iii) and bare glass iv), respectively. These measurements can also be performed with the same particle in a sequential manner. v) Finally, an overpressure of *p*_eject_ = +1000 mbar can be applied to eject the immobilized microgel particle from the aperture. The microgel particles studied here were large enough to follow the whole process by light microscopy, and the corresponding images are shown below the schematic representations. In order to facilitate the optical control, the AFM setup was mounted on an inverted microscope, allowing for the coarse alignment of the aperture and particle and control of the aspiration. It is important to highlight that the sequence i)–v) of particle immobilization, subsequent acquisition of “inverted” indentation measurements, and ejection could be repeated for a large number of microgel particles.

### 2.2 Inverted Nanoindentation by Fluidic Force Microscopy

**Figure 2:**
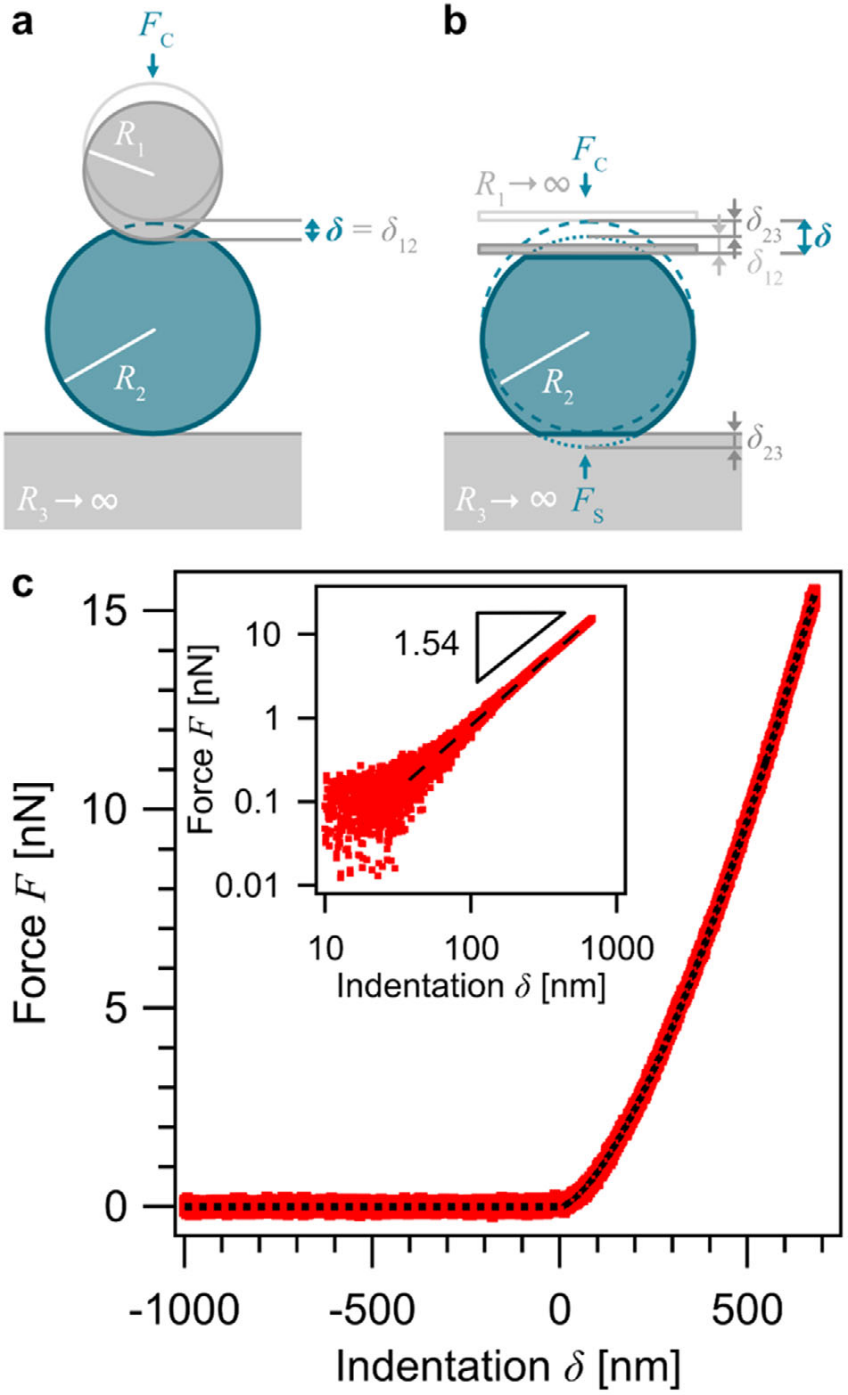
a) Schematic representation of the indentation with a spherical indenter (radius *R*_1_) of a spherical microgel particle (radius *R*_2_) immobilized on a flat substrate (*R*_3_→∞) according to the classical Hertz model. This model provides only an approximate description in the case of small indentations (*δ* ≪ *R*_2_) and large particles (*R*_2_ ≫ *R*_1_). b) Indentation by a flat indenter according to the simplified double contact (SDC) model. The representation follows Glaubitz et al.^[^ ^36^ ^]^ and Raßmann et al.^[^ ^35^ ^]^ Here, the particle has been immobilized on the indenter instead of the substrate (inverted nanoindentation), which is equivalent to classical nanoindentation in the framework of the SDC model. c) Exemplary force versus indentation curve (only approach part) as measured with a POx‐HASH microgel particle by inverted nanoindentation on a bare glass substrate and the corresponding fit (dashed line) according to the SDC model. The inset illustrates in a double logarithmic representation the Hertz power law coefficient of 1.54 fitted to data, which corresponds well to the theoretical value (1.5).

In nanoindentation experiments, the indenter probe is typically ramped into a sample, which is immobilized on a non‐deformable carrier substrate.^[^ ^55^, ^66^, ^67^ ^]^ The most basic model of the resulting elastic response goes back to the work of Heinrich Hertz (cf. **Figure** **2**a).^[^ ^68^ ^]^ However, to provide reasonable results from this model, two conditions must be valid: i) the radius of the sample *R*_2_ must be much larger than the indenter radius *R*_1_ (*R*_2 _≫ *R*_1_,^[^ ^35^, ^36^, ^69^ ^]^ or in the case of films, the indentation depth *δ* must be small compared to the film thickness *h*, i.e., 0.1⋅ *h *≳ *δ* ^[^ ^70^, ^71^ ^]^) and ii) the adhesion between the sample and the indenter must be small and additional surface forces must be negligible.^[^ ^72^, ^73^ ^]^ In the case of the microgel particles that have been studied here, condition i) is clearly not fulfilled. An exception would be indentation by sharp tips with radii in the order of < 50 nm.^[^ ^35^ ^]^ Otherwise, the simplified double contact model has to be used, which also takes the influence of the underlying substrate with the radius *R*_3_ into account for *R*_2 _< *R*_1_.^[^ ^36^ ^]^ In the case of a FluidFM cantilever (cf. Figure 1), this condition is prevalent.

The idea of this study is that the indentation is carried out in an inverted manner compared to classical nanoindentation. Here, the “sample” has been aspirated to a micro‐channeled cantilever and ramped towards the substrate, which also acts as an indenter (cf. Figure 1). Within the framework of continuum contact mechanics, a soft microgel particle attached to a tipless cantilever (cf. Figure 2b) is going to be deformed at both its apex and its bottom when it is pressed against a non‐deformable surface.^[^ ^36^ ^]^ It is irrelevant whether the particle is immobilized on the substrate or the cantilever. The applicability and validity of the SDC model for microgel particles have previously been confirmed experimentally by us^[^ ^35^ ^]^ and other groups.^[^ ^30^, ^74^ ^]^ It should be pointed out that for the Hertz and the SDC model, quasi‐static deformations are assumed, and dynamic as well as hydrodynamic effects are not considered.

Figure 2b illustrates the interaction geometry studied in the following: A spherical particle is compressed between two flat, parallel surfaces. The latter assumption is not strictly valid due to the tilt of the cantilever and will be discussed in detail below. The counteracting forces exerted by the cantilever, *F_C_*, and the substrate, *F_S_*, respectively, act simultaneously on the particle. The SDC model predicts a total deformation δ, which is detected by the AFM nanoindentation experiments. This deformation is the sum of the deformation at the cantilever‐particle contact, δ_12_, and the deformation at the particle‐substrate contact, δ_23_ (cf. Figure 2b). According to the SDC model, one obtains

(1) $$\delta =\delta_{12}+\delta_{23}={\left[\frac{3\left(1-{\nu_{2}}^{2}\right)F}{4E_{2}\sqrt{R_{12}}}\right]}^{2/3}\cdot \frac{1}{k}$$

for the interaction geometry outlined in Figure 2b, namely a particle with radius *R*_2_ between two parallel plates.^[^ ^36^ ^]^ Here, the total deformation is related to the total force *F*, the Poisson ratio ν_2_ and the Young's modulus *E*_2_ of the particle. For the deformation of a sphere between two flat, parallel surfaces, the reduced radius *R*_12_ equals the particle radius *R*_2_ as the radius of the upper plate is infinitely large (*R*_1_ → ∞). A correction factor *k* is introduced for the SDC model that accounts for the used interaction geometry by relating the radii *R*_1_, *R*_2_, and *R*_3_. For our indentation experiments in a plate/sphere/plate geometry (cf. Figure 2b), this correction factor equals *k *= 0.5.

It should be highlighted that this description neglects the influence of the cantilever tilt, which has been examined previously by means of a cantilever with a wedge (parallel plate) and a tipless cantilever (tilted plate with *θ *≃ 10°). For small indentations, such as, ≈ 5% of *R*_2_, no large deviations have been observed.^[^ ^35^ ^]^ Hence, the influence of the tilt angle has been neglected in the following. The influence of adhesion and the applied underpressure during the particle immobilization has been addressed experimentally and will be discussed later.

Figure 2c shows an exemplary force versus indentation curve for the compression of a microgel particle between a micro‐channeled cantilever and a solid substrate. The data shown were acquired using a POx‐HASH microgel particle with *R* ≈ 13.5 µm that has been aspirated to a micro‐channeled cantilever with an aperture of 8 µm in diameter (cf. Figure 1b,c). The immobilized microgel particle has then been ramped towards a planar glass slide, which can be considered as non‐deformable in comparison to the much softer microgel particle, as illustrated by the process outlined in Figure 1d. The dashed line shows the fit to the force versus indentation curve according to the rearranged Equation 1:

(2) $$F\left(\delta \right)=\frac{4}{3}\sqrt{R_{12}}\frac{E_{2}}{1-{\nu_{2}}^{2}}\delta^{3/2}\;k^{3/2}$$

All nanoindentation experiments shown in the following have been performed analogously to the ones reported by Raßmann et al. recently.^[^ ^35^ ^]^ The maximum indentation depth was 5% of the respective particle radius, which has been determined for each particle by light microscopy. The z‐piezo of the AFM was ramped towards the sample at a constant speed of 0.5 µm s^−1^. The inset in Figure 2c shows the force versus indentation curve in a double logarithmic representation. For “inverted” nanoindentation with a PAAm microgel particle, an exemplary force versus indentation curve as well as the Hertz power law coefficient fitted to the logarithmic data is shown in the Supporting Information (cf. Figure S2, Supporting Information).

According to Equations 1 and 2, the SDC model predicts an exponent of 1.5 for the force versus indentation curve independent of the Young's modulus. An experimental exponent of 1.54, determined for the indentation curve shown in Figure 2 was also found as the mean for a larger set of curves originating from both types of hydrogels. Hence, the applicability of the SDC model to describe the indentation process has also been further corroborated for the “inverted” nanoindentation with a FluidFM cantilever.

It should be noted that no significant deviations between the wedge‐shaped cantilevers, which compensate for the cantilever tilt angle as required for the light lever method^[^ ^75^ ^]^ and the tipless cantilevers have been observed.^[^ ^35^ ^]^ Hence, the tilt angle of the FluidFM cantilever has no significant effect on the derived Young's moduli. Hence, the “inverted” nanoindentation setup resembles the parallel plate technique used to study cell mechanics.^[^ ^49^ ^]^

### 2.3 Acquiring Large Nanoindentation Data Sets

**Figure 3:**
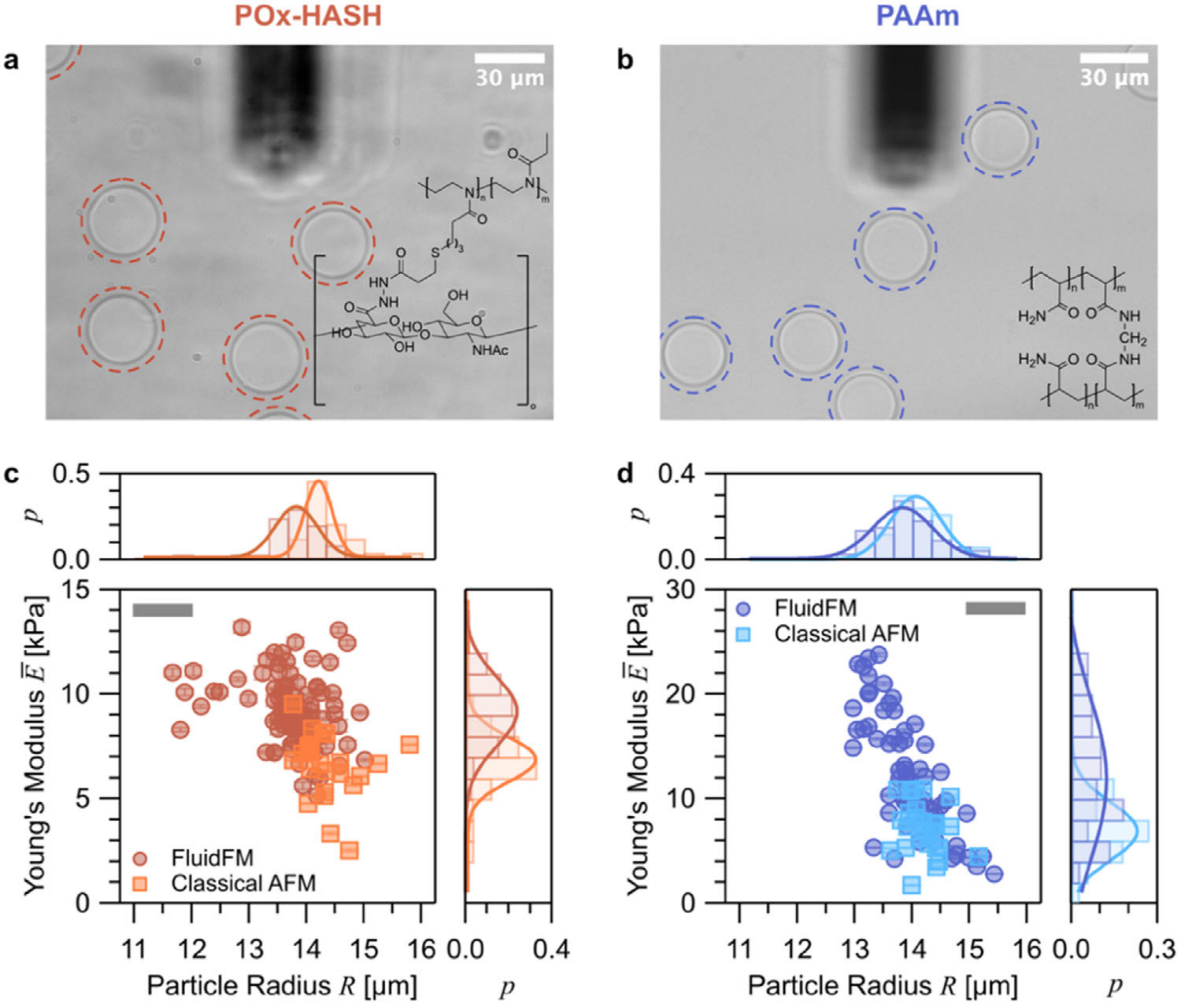
a,b) Bright field microscopy of the microgel particles (POx‐HASH, left and PAAm, right) in the AFM fluid cell. The cantilever is visible at the top of the images. The microgel particles have been indicated by dashed circles. The corresponding chemical structures of POx‐HASH and PAAm, respectively, are shown as insets. c) Scatter plot of the averaged Young's moduli $\bar{E}$ for each particle as a function of the corresponding particle radius *R* for POx‐HASH. Each circular symbol represents the averaged data from one particle. The resulting histograms for the distributions of $\bar{E}$ and *R* are shown on the right and top, respectively. *p* denotes the frequency in the histograms. The Young's moduli have been derived by inverted nanoindentation on bare glass and *p_hold_ *= −100 mbar The square symbols represent data acquired by classical nanonindentation with a spherical colloidal probe (∅ ≈ 6.8 µm) for the same batch of POx‐HASH microgel particles. This data set (squares) has been published recently.^[^ ^35^ ^]^ d) Analogous data set for PAAm microgel particles acquired by inverted nanoindentation on PLL‐*g*‐PEG (circular symbols). For comparison, data obtained by classical nanoindentation for the same batch of PAAm microgel particles is shown additionally (squares).^[^ ^35^ ^]^ All indentation curves have been evaluated by the simplified double contact model with an indentation depth of ≈ 5% of the particle radius. The grey bars illustrate the estimated error in the determination of particle radii by brightfield light microscopy.

In this study, we analyzed the elastic properties of two different microgel systems, namely one based on an ene‐functionalized polyoxazoline crosslinked with thiol‐functionalized hyaluronic acid (POx‐HASH) and one based on polyacrylamide (PAAm). PAAm microgels represent some of the most‐studied hydrogels and microgels.^[^ ^17^, ^30^, ^41^ ^]^ Examples of POx‐HASH and PAAm microgel particles, imaged by light microscopy directly in the AFM setup, are shown in **Figure** **3**a,b, respectively. The corresponding chemical structures are shown as insets. Both hydrogel types comprised particles with radii of 12–16 µm. These relatively narrow size distributions resulted from the microgel syntheses by microfluidics. Further details, as well as a characterization by “classical” nanoindentation, have been reported previously for the very same batches of these microgels.^[^ ^35^ ^]^ Hence, a direct comparison to “classical” nanoindentation experiments was possible. Moreover, utilizing two different types of microgels allowed us to confirm that the here‐proposed approach could be applied universally.

For both types of microgels, i.e. POx‐HASH and PAAm, *n* particles have been analyzed by nanoindentation with a FluidFM cantilever. For POx‐HASH the number of analyzed particles was *n *= 95 and for PAAm it was *n *= 65, respectively. For every particle, the Young's modulus was obtained by independently fitting the force versus indentation data according to the SDC model for a set of at least 30 force curves per particle (cf. Figure 2c). The Young's moduli E were averaged from the fit results for this set of force curves on one particle. The distribution of the mean elastic moduli $\bar{E}$ and their standard deviations σ_*E*_ are summarized in Figure 3c,d as scatterplots, together with the corresponding particle radii as determined by light microscopy, which were also used for the fitting process.

For comparison, we overlaid the data acquired here (i.e., with aspirated microgel particle in the inverted indentation geometry) with data that have been obtained by “classical” nanoindentation.^[^ ^35^ ^]^ For the latter data sets, the nanoindenter was a spherical silica particle with a nominal diameter of 6.8 µm. These data have been reported recently by Raßmann et al. and have been determined on the same microgel batches under the same conditions.^[^ ^35^ ^]^ However, for the “classical” nanoindentation, the microgel particles were immobilized on a bare glass substrate. Furthermore, these data sets were significantly smaller: *n *= 24 for POx‐HASH and *n *= 26 for PAAm, respectively.

Figure 3c shows that for POx‐HASH the Young'moduli $\bar{E}$ fall in the range of 5–14 kPa with a rather large variation. By contrast, the variation on each particle was relatively small, as shown by the error bars. The corresponding distribution of $\bar{E}$ is also shown separately as a histogram on the right side of the graph. The fit to a Gaussian distribution results in a maximum at about 9 kPa. Such broad distributions for microgels have also been reported previously by AFM‐based nanoindentation^[^ ^30^, ^35^ ^]^ as well as other methods.^[^ ^30^ ^]^ An analogous scatter plot for PAAm is shown in Figure 3d with a similarly broad distribution of Young's moduli $\bar{E}$ in the range of 1–23 kPa. Here, a maximum of about 12 kPa has been observed in the corresponding histogram (cf. Figure 3d, right side). The overlaid data obtained by “classical” nanoindentation show very similar distributions for POx‐HASH and PAAm, respectively. Here, the maxima from the Gaussian fit were at about 7 kPa for both particle types.

The observed variances in the elastic moduli between “classical” nanoindentation with a spherical nanoindenter and the inverted nanoindentation with FluidFM are not large but evident. The variation in mean values for the same batch of microgel particles between “classical” nanoindentation with a spherical probe and “inverted” nanoindentation can be attributed to a number of factors. First, the indentation geometry, which is different for the flat FluidFM cantilevers compared to spherical colloidal probes. The same effect has been observed for a spherical indenter and a wedge or tipless cantilever on identical particles.^[^ ^35^ ^]^ We found that for the spherical indenter, a colloid with a diameter of ≈ 6.8 µm, the apparent Young's moduli were about 28% lower for POx‐HASH and 11% for PAAm.^[^ ^35^ ^]^ The here‐observed deviations are in the same order of magnitude as in the previous study. Second, the larger data sets acquired by “inverted” nanoindentation included a broader selection of particle radii, which led not only to a broader distribution but also a shift of the mean values $\bar{E}$.

### 2.4 Influence of Adhesion on the Derived Young's Modulus

**Figure 4:**
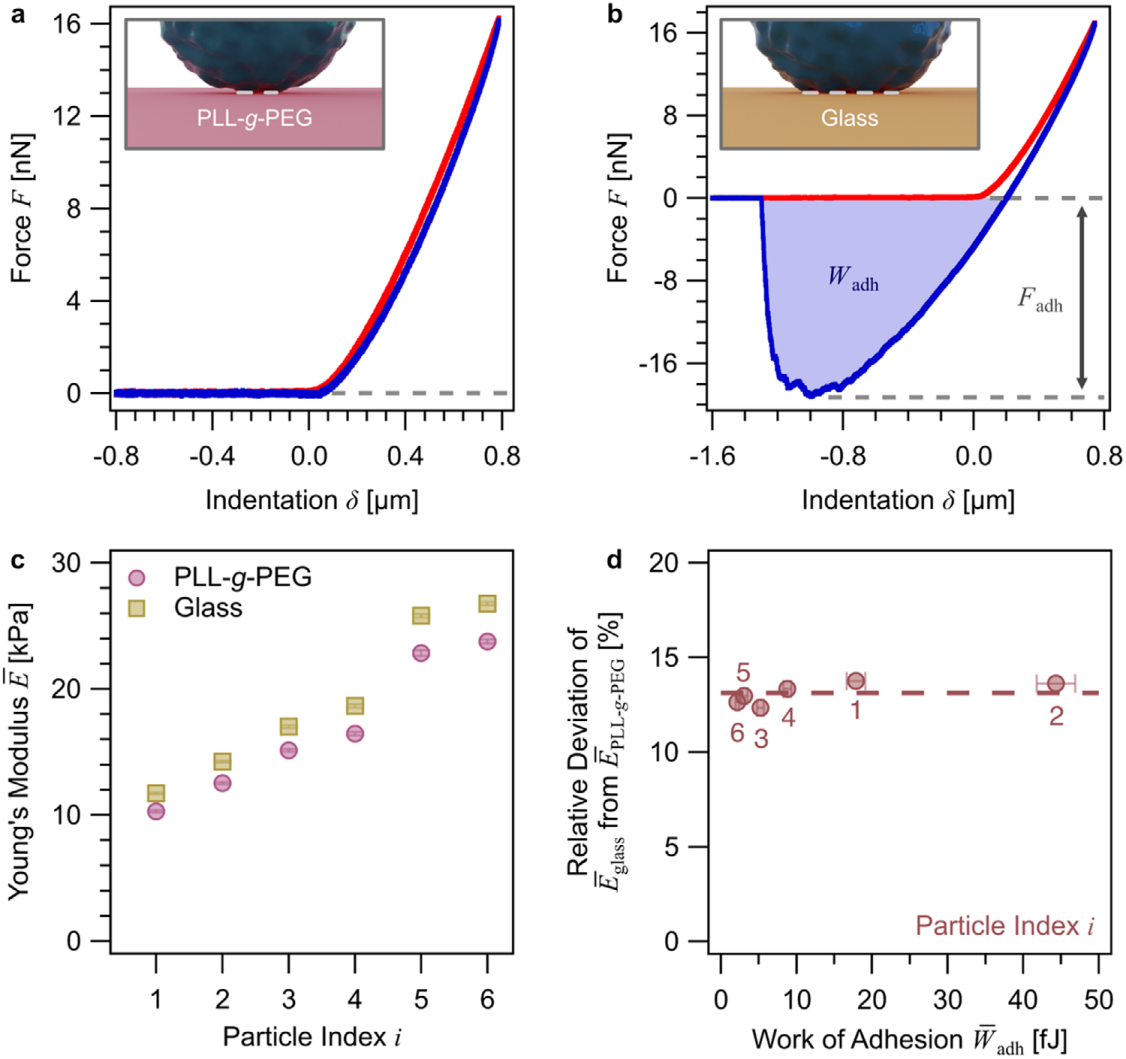
a) Force versus indentation curve for the inverted nanoindentation of a PAAm particle on a PLL‐*g*‐PEG‐functionalized glass surface. The data for approach (red) and retraction (blue) are shown. b) Force versus indentation curve for the inverted nanoindentation of a PAAm particle on bare glass. The adhesion force *F_adh_* and the work of adhesion *W_adh_* are indicated. c) Averaged Young's moduli $\bar{E}$ for six different particles. Each particle *i* has been measured versus a PLL‐*g*‐PEG‐functionalized and a bare glass surface. d) Relative deviation of Young's modulus $\Delta E={\bar{E}}_{\mathrm{glass}}/{\bar{E}}_{\mathrm{PLL}-g-\mathrm{PEG}}$ in percentage as a function of work of adhesion *W_adh_* for each particle.

The FluidFM technique allows for lateral sample movement once a particle is aspirated to the aperture of a micro‐channeled cantilever.^[^ ^63^, ^64^ ^]^ Hence, inverted nanoindentation by FluidFM is the ideal tool to probe the influence of adhesion on the derived Young's moduli. By inverted nanoindentation, it is possible to indent the same particle on different substrates or differently modified areas of one substrate. In the past, differently modified indenters had to be used.^[^ ^72^ ^]^ Thereby, the role of adhesion on the derived Young's moduli can be elucidated in a more unambiguous way for a large ensemble of particles than it would be possible by “classical” nanoindentation. For this study, nanoindentation experiments were consecutively conducted with aspirated PAAm particles on two chemically distinct areas on the same substrate, namely bare and PLL‐*g*‐PEG‐modified areas of a glass slide (cf. Figures 1d and 4). An analogous experiment for the indentation of POx‐HASH microgel particles is shown in the Supporting Information (cf. Figure S3, Supporting Information).

Exemplary force versus indentation curves on both surface chemistries, bare and PLL‐*g*‐PEG‐modified glass, are shown in **Figure** **4**a,b, respectively. The corresponding force versus distance curves are shown in the Supporting Information (cf. Figure S4, Supporting Information). The data in Figure 4 have been acquired with an aspirated PAAm microgel particle. Here, we show the data acquired during approach and subsequent indentation of the microgel particle (red data in Figure 4a,b) as well as the data acquired during retraction (blue data in Figure 4a,b). The latter data allowed us to estimate the adhesive behavior during the separation of the aspirated particle from the substrates. The maximum force applied is often referred to as the adhesion force *F*_adh_.^[^ ^76^ ^]^ Moreover, we additionally determined the work of adhesion *W*_adh_, which corresponds to the work required to completely separate the particle from the substrate.^[^ ^76^ ^]^ In the case of easily deformable microgel particles and in the absence of a clear snap‐off, the work of adhesion generally provides a better description for the strength of adhesion.^[^ ^77^ ^]^ The adhesion data shown here were always acquired for all particles on both types of substrates. In the case of PAAm microgels, these were *n *= 6 different particles (for POx‐HASH, see Figure S3, Supporting Information).

For both microgel types studied here, practically no adhesion could be detected when retracting PAAm and POx‐HASH microgel particles from a PLL‐*g*‐PEG‐functionalized surface (cf. Figure 4a; Figure S3a, Supporting Information, respectively). This finding is in line with previous reports on PLL‐*g*‐PEG‐functionalized surfaces.^[^ ^78^, ^79^ ^]^ PLL‐*g*‐PEG is a random graft copolymer with a poly(l‐lysine) backbone and poly(ethylene glycol) side chains. The latter, i.e. PEG, is well‐known as an anti‐adhesion coating for cells and bacteria and leads generally to low adhesion,^[^ ^78^ ^]^ while the l‐lysine serves as an anchor to the substrate.^[^ ^79^ ^]^ Please notice that also the FluidFM cantilever has been modified by the same copolymer, i.e. PLL‐*g*‐PEG, in order to allow for a reversible attachment of microgel particles to the micro‐channeled cantilever. By contrast, on bare glass, significant adhesion has been observed for both types of microgels (cf. Figure 4b; Figure S3b, Supporting Information, respectively). However, for both surface chemistries, the hysteresis in the compression region (i.e., during approach and *F *> 0) of the force versus indentation curves was small, indicating an elastic deformation regime.

Figure 4c summarizes the Young's moduli derived for several PAAm particles (*n *= 6) from inverted nanoindentation on both surfaces. For each particle, we determined the Young's moduli by fitting the SDC model to the approach curves of 30 force versus indentation curves. It has to be pointed out that the SDC model does not include any adhesive processes. The mean elastic modulus $\bar{E}$ for each particle *i* is shown in Figure 4c. The particles have been sorted by their ascending Young's modulus ${\bar{E}}_{glass}\left(i\right)$ as determined for the bare glass surface. The moduli on glass varied between 10 and 28 kPa. Thus, falling in the range observed independently for a much larger data set (cf. Figure 3d). We observed for all particles studied that the Young's modulus derived from the data on bare glass was higher than that on the PEG‐coated glass (i.e., ${\bar{E}}_{glass}\left(i\right)$ > ${\bar{E}}_{\mathrm{PLL}-g-\mathrm{PEG}}\left(i\right)$). In the Supporting Information, we demonstrated that for the example of POx‐HASH that this relation did not depend on the order in which the surfaces had been measured. Our findings are in line with the simple assumption that large adhesion leads to a “pre‐stress” and thus a deformation of the particle without any external force. The relative deviation between ${\bar{E}}_{glass}\left(i\right)$ and ${\bar{E}}_{\mathrm{PLL}-g-\mathrm{PEG}}\left(i\right)$ was relatively constant at about 13% for each particle and independent of the corresponding mean work of adhesion ${\bar{W}}_{adh}\left(i\right)$ for this particle on bare glass. These data have been summarized in Figure 4d. The observed independence of the relative deviation from ${\bar{W}}_{adh}\left(i\right)$ indicates most likely a strong correlation between the contact area and the elastic modulus $\bar{E}\left(i\right)$ for each particle *i*. A similar relation is commonly used to determine the work of adhesion from the contact radius, as determined by light microscopy.^[^ ^73^ ^]^ In the next paragraph, we will examine the contact radius forming between a microgel particle and the two substrates without any external forces acting.

### 2.5 Characterization of Adhesion on Different Substrates

Adhesion between a soft particle and a substrate leads to a finite contact area and, thus, a partial deformation of the particle even when no external forces are acting, which is predicted by continuum theories like the one of Johnson, Kendall, and Roberts (JKR)^[^ ^80^, ^81^ ^]^ and has also been experimentally verified.^[^ ^82^, ^83^ ^]^ Due to the exerted pre‐stress, the adhesive behavior affects the determination of the Young's modulus by AFM‐based nanoindentation experiments if not accounted for, as we demonstrated in the previous paragraph.^[^ ^35^ ^]^ In the following, we visualized and determined the difference in the pre‐stress on two substrates (bare and PLL‐*g*‐PEG‐modified glass) by a surface‐sensitive light microscopy technique. Here, total internal reflection fluorescence (TIRF) microscopy was used, which makes use of the evanescent field formed under total reflectance to excite fluorophores at the glass interface.^[^ ^84^ ^]^ Therefore, the PAAm microgel particles utilized in these experiments were synthesized from fluorescein‐labeled PAAm (FITC‐PAAm). Transmitted light microscopy has been used for determining the radius *R* of the microgel particles.

**Figure 5:**
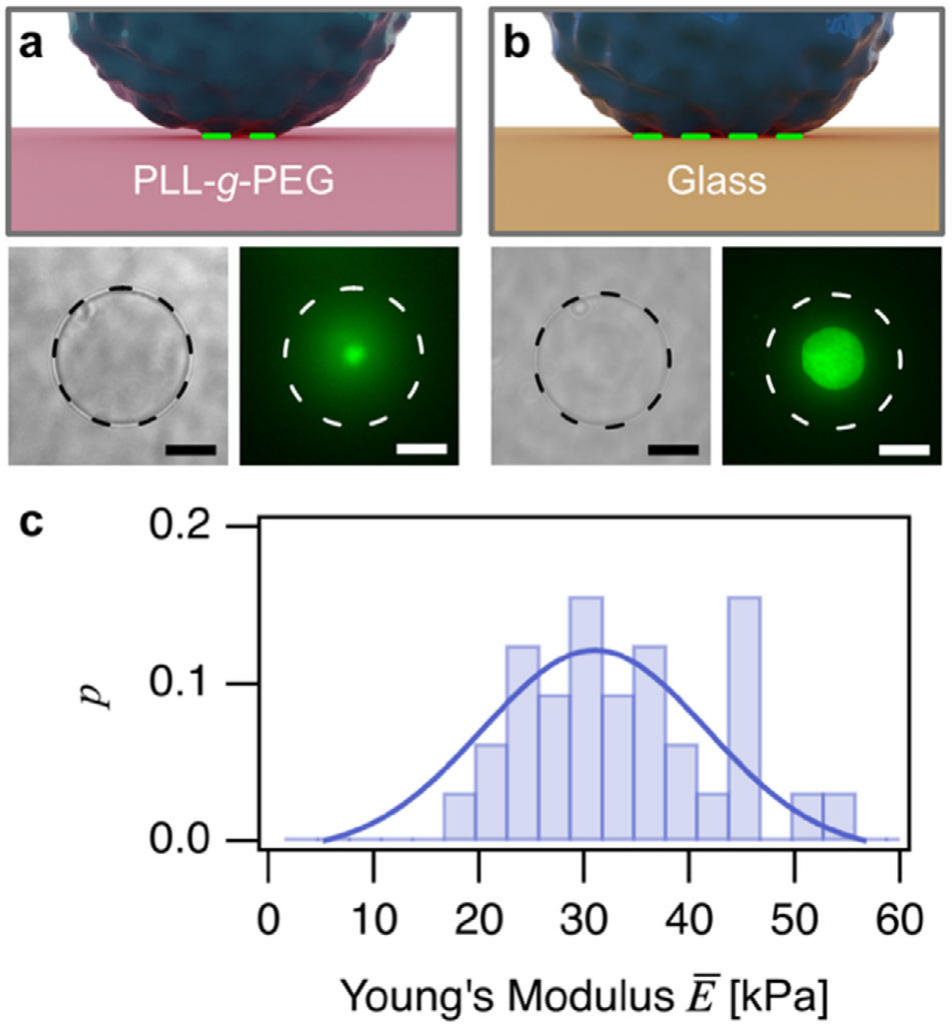
Schematic representations of different contact areas of a microgel particle on a) PLL‐*g*‐PEG‐functionalized glass and b) bare glass, respectively. Brightfield optical microscopy (bottom left) and total internal reflection fluorescence microscopy (TIRF) (bottom right) of FITC‐PAAm particles on a) PLL‐*g*‐PEG‐functionalized glass and b) bare glass. The dashed circles indicate the corresponding particle diameters (black and white, respectively). The green fluorescence signal corresponds to the contact areas in the TIRF‐images. The scale bars correspond to 10 µm. c) Distribution of Young's moduli for FITC‐PAAm microgel particles (*n *= 32) as determined analogously to the ones for non‐labelled PAAm microgels (cf. Figure 3b).

**Figure** **5**a and b show in a schematic manner the expected variation of the contact area of the microgel particle and, thus, the resulting pre‐stress on PLL‐*g*‐PEG modified glass (cf. Figure 5a top) and on bare glass (cf. Figure 5b top). Below, the corresponding experimental results, namely brightfield optical microscopy (bottom left) and TIRF images (bottom right), for the two different surface chemistries and exemplary FITC‐PAAm microgels are depicted. It has to be highlighted that these TIRF images were acquired for adsorbed microgel particles without any externally applied force. It is evident that for the PLL‐*g*‐PEG‐coated surfaces, the contact area was much smaller than for the bare glass, therefore indicating a reduced adhesion on PEG in comparison to bare glass surfaces, corroborating the results from the direct force measurements (cf. Figure 4a,b). A quantitative evaluation shows that the contact radius on glass *R*_cont_ was 6.3 ± 0.7 µm while on PLL‐*g*‐PEG it was 3.1 ± 1.2 µm (cf. Figure 5a,b bottom right). By comparison, the particle diameters were nearly comparable with 26.7 µm (cf. Figure 5a) and 27.0 µm (cf. Figure 5b) as determined by brightfield light microscopy for both particles. In analogy to a recent study, we also reported the ratio of *R*_cont_/*R* for both types of surfaces, with 0.23 ± 0.09 for PLL‐*g*‐PEG and 0.47 ± 0.05 for glass (cf. Figure S5, Supporting Information).^[^ ^35^ ^]^ Please notice that compared to Raßmann et al.,^[^ ^35^ ^]^ we used a slightly different data evaluation procedure in order to allow for a comparison between the adhesion on bare and modified glass (cf. Supporting Information).

The FITC‐labelling of PAAm microgel particles did lead to higher Young's moduli, as the particle synthesis had to be slightly adapted by copolymerization of APMA. The distribution of Young's modulus for *n *= 32 FITC‐PAAm particles is shown in Figure 5c and has been determined analogously to the data obtained for the non‐labeled PAAm microgels (cf. Figure 3b). Both types of PAAm microgels had a similar size distribution of 12–14 µm for FITC‐PAAm versus 13–15 µm for unlabeled PAAm. The TIRF data for the adsorbed particles support the assumption of a significantly larger pre‐deformation due to the increased adhesion for the particles on bare glass. The TIRF data agree with the larger adhesion forces and work of adhesion detected on the glass surface and corroborate the interpretation of the ≈ 13% larger derived elastic modulus as resulting from the pre‐deformation due to adhesion.

### 2.6 Influence of the Holding Pressure on the Derived Young's Modulus

**Figure 6:**
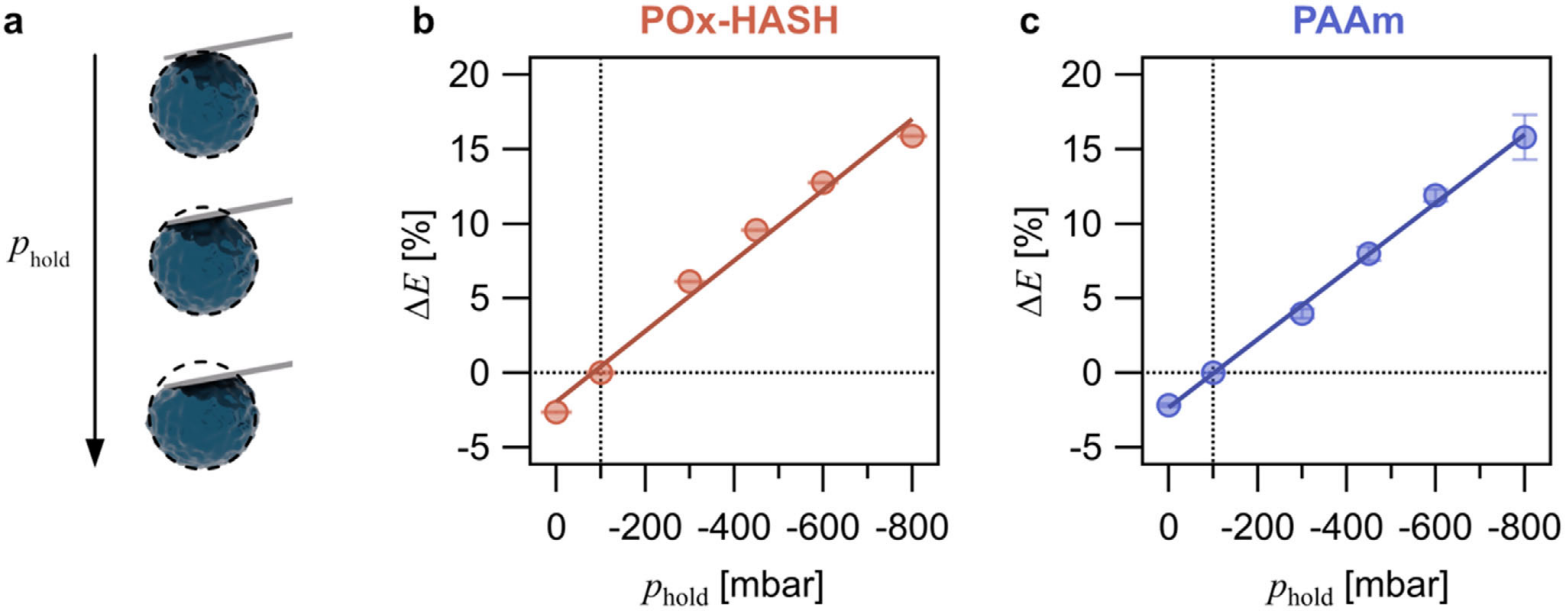
a) Schematic representation of particle deformation by increasing holding pressures *p_hold_*. b) Influence of the holding pressure on the Young's modulus for POx‐HASH microgel particles. Change of the derived Young's modulus as a function of holding pressure. Here, the relative deviation ∆*E* in percentage of the Young's modulus $\bar{E}$ in relation to the value obtained at *p_hold_ *= −100 mbar is reported. c) Analogous experimental data for PAAm.

The role of pre‐deformation has also been studied by a different approach, which was based on the principle of fluidic force microscopy. In order to immobilize particles at the aperture, an underpressure has been applied, the so‐called holding pressure *p*_hold_ (cf. Figure 1d). This pressure *p*_hold_ is supposed to lead to a deformation of the soft microgel particles as schematically shown in **Figure** **6**a. Larger holding pressures *p*_hold_ will lead to larger deformations. The resulting effect would be similar to adhesive behavior, with the difference that the adhesion can be tuned *in situ*. In the conceptual framework of the SDC model, adhesion to both contact areas will be comparable. In the following, we will corroborate this hypothesis experimentally for both types of microgels by probing “inverted” nanoindentation against a PLL‐*g*‐PEG‐functionalized glass surface and varying the holding pressure. The holding pressure has been adjusted by means of the external microfluidic controller. Here, we varied the holding pressure between 0 and −800 mbar. Please notice that for all experiments presented before, we used a constant holding pressure of *p*_hold_ = −100 mbar.

Figure 6b,c shows the relative deviation of the Young's moduli from the one derived at *p*_hold_ = −100 mbar as a function of *p*_hold_. The derived Young's moduli $\bar{E}$(*p*_hold_) have been determined by the identical procedure used for Figures 3c,d and 5c. For both POx‐HASH and PAAm microgel particles, we find a monotonic increase of the derived Young's moduli $\bar{E}$(*p*_hold_) from *p*_hold_. As indicated by the schematic representation in Figure 6a, it was no surprise that an increasing *p*_hold_ leads to an increase in the derived elastic moduli. This finding is in line with recent simulations ^[^ ^35^ ^]^ where the apparent Young's modulus was increasing with a stronger adhesion to the underlying substrate. However, the nearly perfectly linear dependence of the deviation and an identical slope for both microgel types (cf. Figure S6, Supporting Information) was somehow unexpected. We expected that those slopes would differ due to different elastic moduli of both hydrogels and the difference in the adhesion behavior, as also the prestress exerted as a function of the external aspiration would be different. The observed dependence on the aspiration should be further followed up by finite element simulations. It should also be kept in mind that hydrogels are primarily composed of water, and similar deformations have been observed by micropipette aspiration.^[^ ^85^ ^]^ The linear relation of derived Young's modulus and the applied aspiration pressure holds some important conclusions. Even the moderate pressure of *p*_hold_ = −100 mbar did led to an overestimation of the Young's moduli of about 2.7% for POx‐HASH and 2.2% for PAAm, respectively, compared to the corresponding elastic modulus at 0 mbar. Therefore, the aspiration pressure should be set as small as possible to prevent an overestimation of the Young's modulus. Thereby, the influence of the holding pressure on the derived Young's moduli has been minimized. It should be noted that the FluidFM cantilever has been modified by PLL‐*g*‐PEG in order to decrease adhesion with the microgel. However, the TIRF data indicate that even for a PEG‐modified surface, the contact radius was finite under zero load. The here‐observed behavior can be compared to that for solid particles, where no difference in the force versus distance curves in dependence of the applied aspiration pressures was found.^[^ ^62^, ^64^ ^]^ Hence, fluid dynamics does not influence the interaction forces but primarily the adhesion.

### 2.7 Towards Higher Throughput

The FluidFM technique allowed for a comparatively fast mechanical characterization of microgel particles by “inverted” nanoindentation. Its effectiveness results primarily from the simple and fast aspiration of particles. The most time‐consuming step of “classical” nanoindentation, namely the fine centering of the indenter above the particle, can thereby be completely avoided. However, other manipulation steps, like positioning the cantilever over a particle and controlling successful aspiration by light microscopy, still have to be performed manually under the supervision of an operator. In the following, we outline how these steps might be automated in the future in order to sample an even larger number of microgel particles by “inverted” nanoindentation with fluidic force microscopy.

**Figure 7:**
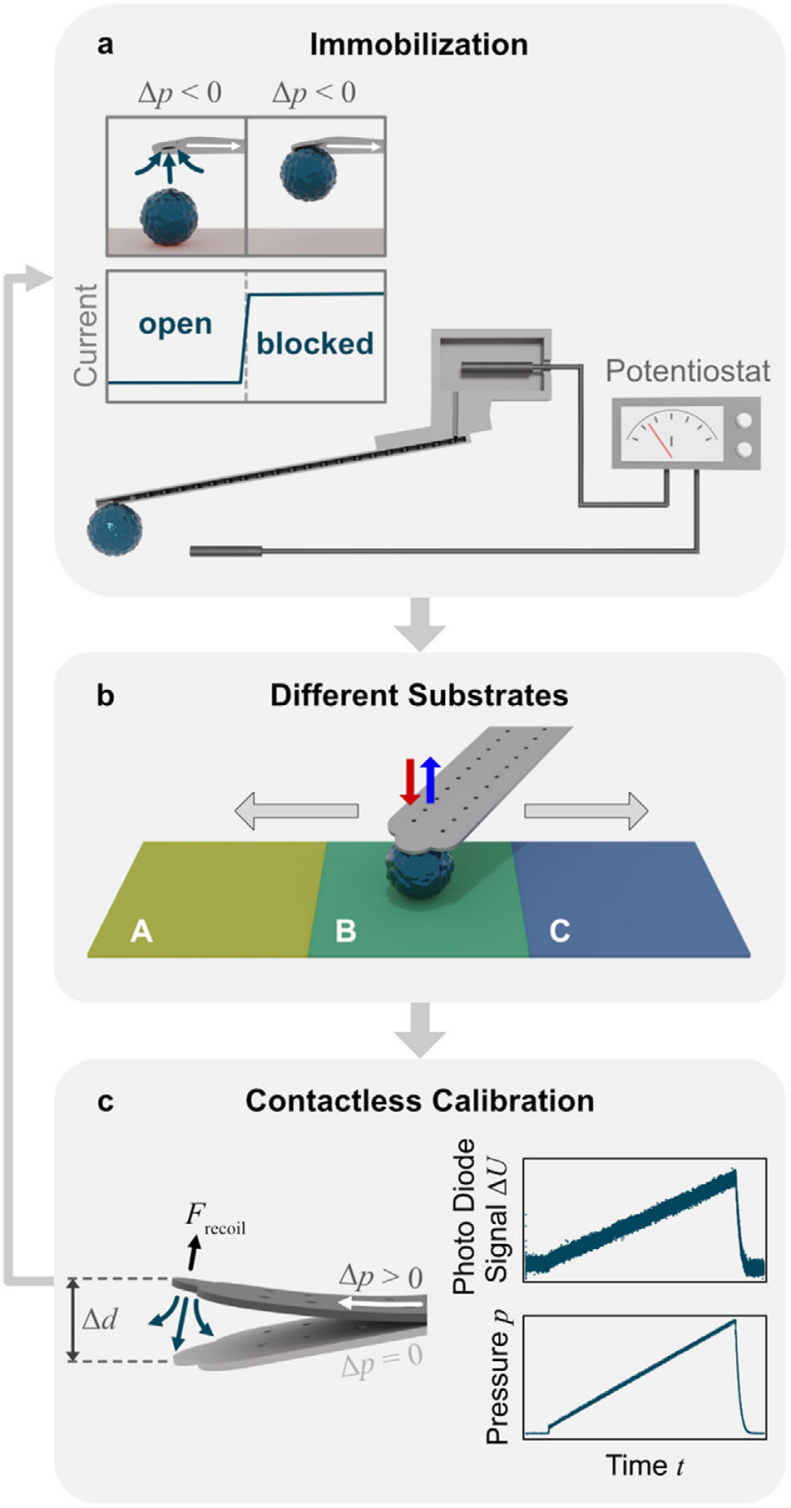
Schematic representation of an automation process for inverted nanoindentation by fluidic force microscopy. a) Electrokinetic detection of particle immobilization at the aperture by monitoring the current profile through the internal channel of the cantilever.^[^ ^65^ ^]^ b) Measurement with an aspirated particle against different surfaces to determine adhesion and Young's modulus, respectively. c) Contactless calibration of the inverted optical lever sensitivity (InvOLS) is required to determine the acting forces. The shown method is based on the recoil effect when an overpressure is applied to the internal micro‐channel of the cantilever.^[^ ^86^ ^]^ Repetition of steps a)–c) enables fully automated and unattended measurements that would allow the collection of large data sets.

The incorporation of an independent detection mechanism for successful aspiration of particles would allow to overcome an important requirement for human supervision during the current experiments. Particle immobilization at the aperture has been controlled here visually by optical microscopy (cf. Figure 1e). An alternative, easily automatable detection mechanism is based on current/potential measurements over the micro‐channel:^[^ ^65^ ^]^ During the particle aspiration, a constant aspiration pressure of *p_asp_ *= −800 mbar has been applied. The resulting streaming of liquid through the internal channel results in a potential difference between two electrodes, one in the liquid cell and one in the reservoir of the micro‐channeled cantilever. This streaming potential can be acquired throughout the aspiration process. Blocking of the aperture by a particle leads to a rapid drop in the detected potential. This approach has been presented already and is schematically shown in **Figure** **7**a.^[^ ^65^ ^]^ We could also demonstrate that the streaming potential detection works with hydrogel particles despite their significantly high water content.^[^ ^65^ ^]^ After the successful aspiration of a particle, direct force measurements can be performed on various substrates bearing different surface functionalities (cf. Figure 7b), analogously to what is demonstrated here for two substrates (cf. Figure 4).

For longer measurements, it is important to keep track of instrumental drifts, especially about the inverted optical lever sensitivity (InvOLS).^[^ ^75^ ^]^ The InvOLS has a significant quantitative influence on the conversion of the raw data into force versus indentation curves.^[^ ^76^, ^86^, ^87^ ^]^ Recently, new approaches for contact‐free calibration of the InvOLS in the case of micro‐channeled cantilevers have been presented.^[^ ^86^, ^88^ ^]^ The approach of Sittl et al. makes use of the recoil building up while liquid is pressed through the aperture (cf. Figure 7c).^[^ ^86^ ^]^ For an automated procedure, keeping track of this recoil would also be important to identify full or partial clogging of the aperture caused by debris during particle aspiration.

The proposed fully automated workflow for “inverted” nanoindentation using the FluidFM technique is summarized in Figure 7. The three steps include: 1) detection of the particle immobilization, 2) performing adhesion measurements as well as inverted nanoindentation on various surfaces, and 3) calibrating the cantilever's InvOLS. These steps can be repeated in principle as often as required for a large number of hydrogel particles, making not only high‐throughput but also unattended FluidFM measurements possible to create large data sets. The here studied “inverted” nanoindentation represents the base of the proposed workflow. However, it has to be stated that this workflow has not been implemented yet, which was also not within the scope of this study.

## 3 Conclusion

“Inverted” nanoindentation by aspiration of microgel particles to micro‐channeled cantilevers is a novel approach to the mechanical characterization of particulate soft matter. Despite the availability of other characterization techniques,^[^ ^51^, ^53^ ^]^ nanoindentation still represents a “gold standard” for the determination of Young's moduli for micrometer‐sized objects. Our new approach can overcome one of the major restrictions, namely the possibility of studying only a very limited number of particles by “classical” nanoindentation. Especially in the case of microgels, even when prepared by microfluidics, the Young's moduli vary significantly within one batch. Hence, the acquisition of a statistically reliable data set is of utmost importance to fully characterize whole microgel batches. By varying the size of the aperture, smaller particles can also be studied. Cantilevers with a higher spring constant will also enable the indentation of particles with Young's moduli up to an order of ≈ 500 kPa that are much more rigid than the microgel particles studied here. However, currently the FluidFM cantilevers are only commercially available with spring constants *k_c_* of up to 4 N m^−1^, which will nevertheless be stiff enough for most soft materials. While the sandwiched structure of the FluidFM cantilevers requires a sophisticated production process, they are commercially available, albeit more expensive than “standard” cantilevers. Nevertheless, their internal structure does not affect the accuracy of the indentation measurements once the spring constant has been determined.^[^ ^86^, ^88^ ^]^

The simplified double contact (SDC) model^[^ ^35^, ^36^ ^]^ was essential for quantitative analysis of the data from “inverted” nanoindentation, where the particle is aspirated and pressed against a fixed substrate. The SDC model also provides a natural framework to describe the geometrical constraints for the “inverted” nanoindentation approach. We could confirm the results reported recently for the same batches of two different microgels, namely POx‐HASH and PAAm, by “classical” AFM‐based nanoindentation. Moreover, a new approach to further elucidate the role of adhesion has been enabled, as it was possible to determine the Young´s moduli on different surfaces. The same effect as seen for surface‐based adhesion could be induced by means of the externally applied aspiration pressure. Thereby, the recently postulated role of adhesion for the apparent Young's moduli could be verified independently.

Our approach can be further developed by automating the remaining manual manipulation steps. Thereby, even larger data sets can be acquired by unattended measurements. The advent of robotic approaches based on fluidic force microscopy has been pursued previously,^[^ ^86^ ^]^ but not about the mechanical response of particulate or cellular samples. While some techniques, such as the micropipette technique, can also be automatized in combination with optical microscopy and automatic image analysis,^[^ ^34^, ^89^ ^]^ the “inverted” nanoindentation allows the study of the influence of adhesion by varying the substrate against which the indentation is carried out. Adhesion has a large influence on the derived Young's moduli, and thus extracting its influence allows for a more accurate determination of the Young's moduli of soft particulate matter. Such measurements will enable new perspectives, especially about the role of the substrates involved, and allow for the simultaneous characterization of mechanical response and adhesion. We believe that such data sets will become increasingly important with the advent of granular hydrogels. Moreover, the here‐described approach can also be applied to cells and bacteria, where the distribution of mechanical properties can be even broader. However, it should be kept in mind that for “inverted” nanoindentation, a spherical shape of the sample is a prerequisite in order to apply the SDC model.^[^ ^36^ ^]^

## 4 Experimental Section

### Materials

All aqueous solutions were prepared with water of Milli‐Q quality (resistivity of 18 MΩ cm^−1^ at 25 °C, IQ7000, Merck KGaA, Darmstadt, Germany). Krytox 157 FSH (H. Costenoble GmbH & Co. KG, Eschborn, Germany), methanol (≥99.9%, Fisher Scientific, Loughborough, UK), ethanol (p.a., VWR Chemicals, Darmstadt, Germany), *n*‐hexane (VWR International, Radnor, USA), HFE 7100 and HFE 7500 (IOLITEC GmbH, Germany), ammonia 25% (VWR International S. A. S, Rosny‐sous‐Bois, France), acrylamide (AAm, ≥99%, Sigma–Aldrich, St. Louis, USA), *N,N′*‐methylenbis(acrylamide) (BIS, 99%, Sigma–Aldrich, St. Louis, USA), *N*‐(3‐aminopropyl)methacrylamide hydrochloride (APMA, 98%, Sigma–Aldrich, St. Louis, USA), ammonium persulfate (APS, 98%, Sigma–Aldrich, St. Louis, USA), *N,N,N′,N′*‐tetramethylethylenediamine (TEMED, ≥99%, Sigma–Aldrich, St. Louis, USA), 2‐propanol (p.a., VWR Chemicals, Darmstadt, Germany), phosphate buffered saline (1×PBS, pH 7.4, Sigma–Aldrich, St. Louis, USA), fluorescein 5‐isothiocyanate (FITC, ≥90%, Cayman Chemical Company, Ann Arbor, USA), Hellmanex III (Hellma GmbH & Co. KG, Müllheim, Germany), hydrogen peroxide (30 w/v%, Fisher Scientific, Schwerte, Germany), mineral oil (Sigma–Aldrich, Darmstadt, Germany), Span 80 (Sigma–Aldrich, Darmstadt, Germany), lithium‐phenyl‐2,4,6‐trimethylbenzoylphosphinate (LAP, TCI Chemicals, Eschborn, Germany), 1H,1H,2H,2H‐perfluoro‐1‐octanol (PFO, 97%, Sigma–Aldrich, St. Louis, USA), poly(L‐lysine)‐*graft*‐poly(ethylene glycol) (PLL‐*g*‐PEG, Susos AG, Dübendorf, Switzerland), circular glass discs (diameter of 35 mm, thickness of 1 mm, Irlbacher Blickpunkt Glas GmbH, Schönsee, Germany) and circular glass cover slips (diameter of 10 mm, thickness of 0.13–0.16 mm, Menzel GmbH & Co. KG, Braunschweig, Germany) were used as received.

### Surfactant Synthesis

As surfactant, the ammonium carboxylate salt of Krytox 157 FSH was synthesized according to a modified procedure previously published by Girardo et al.^[^ ^30^ ^]^ Krytox 157 FSH (10 g) was completely dissolved in methanol (60 mL) and HFE 7100 (30 mL). After the dropwise addition of ammonia (25 mL, 0.1 m), the mixture was stirred overnight while the reaction took place. The solvent was then removed by means of a rotary evaporator (Laborota 4000 efficient, Heidolph Scientific Products GmbH, Schwabach, Germany). The product was dried overnight at 80 °C under vacuum. A pale, viscous oil was obtained and stored at room temperature.

### Microfluidic Synthesis of PAAm Particles

Polyacrylamide hydrogel (PAAm) particles were synthesized by droplet‐based microfluidics, following the description of Girardo et al.^[^ ^30^ ^]^ with marginal modifications. A mixture of the monomers acrylamide (AAm) and bis‐acrylamide (BIS) with a BIS/AAm ratio of 3.25% (w/w) and a total monomer content of 18.6% (w/v) was prepared by dissolving in Milli‐Q water. The monomer solution as well as pure Milli‐Q water were purged with argon for 30 min. Then, ammonium persulphate (APS) was added to the purged water at a concentration of 16 g L^−1^.

FITC‐labeled PAAm particles (FITC‐PAAm) were synthesized according to a modified procedure as described by Hu et al.^[^ ^90^ ^]^ Contrary to the fabrication of bare PAAm particles, *N*‐(3‐aminopropyl)methacrylamide hydrochloride (APMA) was used in place of 5 mol% of AAm. A mixture of surfactant (1.5% (w/w)) and *N,N,N′,N′*‐tetramethylethylenediamine (TEMED) in HFE 7500 (0.4% (v/v)) served as an oil phase. No argon purging has been done here.

The fabrication of the microfluidic device is described in more detail in the Supporting Information (cf. SI). PEEK‐tubing (1/32″ OD × 0.010″ ID, VICI AG International, Schenkon, Switzerland) was used to connect the microfluidic device to two 2 mL vials acting as reservoirs. One of the vials was filled with the oil phase (1 mL) and the other one with a mixture of the monomer solution (300 µL) and the aqueous APS solution (300 µL). The latter had been gently mixed beforehand to prevent oxygen dissolution. Flow EZ pressure controllers (Fluigent S.A., Le Kremlin‐Bicêtre, France) were utilized to set the reservoirs under pressure. A controlled droplet formation was enabled by using Flow Unit S flow sensors (Fluigent S.A, Le Kremlin‐Bicêtre, France). Specific flow rates were applied for the aqueous phase (300 µL h^−1^) and the oil phase (750 µL h^−1^), respectively. The droplet formation was followed by optical light microscopy (AxioObserver, Carl Zeiss AG, Oberkochen, Germany), using a high‐speed camera system (DeCellerator, Zellmechanik Dresden GmbH, Dresden, Germany) with framerates up to 10,000 fps. Finally, the droplets were gathered in a vial filled with 500 µL of the oil phase. The collection of droplets was stopped 20 min after the monomer and APS solutions were mixed.

After shaking the resulting emulsion overnight at 20 °C (800 rpm), the clear oil phase was allowed to settle and was then removed. An emulsion breaker (500 µL, 20% (v/v) PFO in HFE 7500) was added, followed by vortexing (10 s) and centrifuging (5000 g, 60 s) the mixture. After removal of the oil phase, *n*‐hexane (500 µL) was added. The mixture was vortexed (10 s) and centrifuged (5000 g, 60 s) again. *n*‐Hexane was removed, and phosphate‐buffered saline (1×PBS, 100 µL for bare and 300 µL for APMA‐modified particles) was added. The resulting particle suspension was stored in the refrigerator at 4 °C. Less concentrated particle suspensions were obtained by diluting the stock suspension with 1×PBS.

### FITC‐Labeling of PAAm Particles

The stock suspension of APMA‐modified particles (150 µL) and a solution of fluorescein 5‐isothiocyanate in 1×PBS (2 mL, 0.1 mg mL^−1^) were mixed and shaken overnight at 20 °C (800 rpm). After centrifugation (8000 g, 120 s) of the suspension, the residual particles were resuspended in 1×PBS (1.5 mL). This centrifugation and resuspension sequence was repeated six times. The particles were resuspended in 1×PBS (500 µL) after the last centrifugation and stored in the refrigerator at 4 °C.

### Microfluidic Synthesis of POx‐HASH Particles

POx‐HASH particles were synthesized from ene‐functionalized polyoxazoline (POx) and thiol‐functionalized hyaluronic acid (HASH) as described elsewhere.^[^ ^91^, ^92^ ^]^ For the pre‐gel solution, POx (3.75% (w/v)) and HASH (3.75% (w/v)) were dissolved in 1×PBS buffer at pH 7.4, providing a solution with a total polymer content of 7.5% (w/v). Lithium‐phenyl‐2,4,6‐trimethylbenzoylphosphinate (1% (w/v)) was added as photo‐initiator for photo‐crosslinking. The polymer solution was filtered through a Target2 PTFE syringe filter (17 mm diameter, 0.2 µm pore size, Thermo Fisher Scientific Inc., Waltham, USA) before use. A mixture of mineral oil and Span 80 (2.5% (w/v)) was used as the oil phase. Glass syringes (1x SYR 1 mL 1001 TLL for the aqueous phase, 2x SYR 5 mL 1005 TLL for the oil phase, Hamilton Bonaduz AG, Bonaduz, Switzerland) loaded with the mixed precursor solutions were mounted in displacement‐based syringe pumps (neMESYS, Cetoni GmbH, Korbussen, Germany). The glass syringes were connected to PE tubes (I.D. 0.28 mm, O.D. 0.64 mm, Scientific Commodities Inc., Lake Havasu City, USA) via precision tips (TIP 30GA 0.006X.25, Nordson EFD, West Lake, USA) utilizing Luer‐Lock connections. The PE tubings were linked to the inlet chambers of the microfluidic device. Details on the fabrication of the microfluidic device are given in the Supporting Information (cf. SI). The oil phase was connected to the two outer inlets and the polymer phase to the middle inlet. The QmixElements software (Cetoni GmbH, Korbussen, Germany) was used to adjust the liquid flow by controlling the syringe pumps. Defined flow rates were applied for the oil phase (460 µL h^−1^ each, 920 µL h^−1^ in total) and the aqueous phase (48 µL h^−1^). The droplet formation was followed by optical light microscopy (AxioVert.A1 FL, Carl Zeiss AG, Oberkochen, Germany) using a high‐framerate video camera (Phantom v1610, Vision Research Inc., Charlottetown, Canada). The resulting droplets were transferred to a PE tubing that was linked to the outlet chamber of the microfluidic device. Here, the droplets were crosslinked via a photo‐initiated thiol‐ene click reaction by exposing them to a 405 nm power LED (Silver‐LED‐405, Fiber Coupled UV, Prizmatix, Holon, Israel) for 5 s. A 10 mL glass vial was used for collecting the resulting microgel particles. The POx‐HASH particles in the oil phase were washed two times by centrifuging (11500 g, 150 s) with *n*‐hexane and three times by centrifuging (11500 g, 150 s) with 1×PBS. The resulting aqueous particle suspension was stored in the refrigerator at 4 °C.

Further information on the preparation of the microfluidic chips used in the synthesis can be found in the supporting information (cf. Section S1, Supporing Information).

### Substrate Preparation

Circular glass discs with a diameter of 35 mm were used as substrates and cleaned according to a modified RCA cleaning routine.^[^ ^93^ ^]^ At first, the circular glass discs were sonicated in an aqueous Hellmanex III solution (2% (v/v)) at 40 °C for 20 min. After thoroughly rinsing with Milli‐Q water, the glass discs were sonicated in a mixture of 2‐propanol and Milli‐Q water (3:1 (v/v)) at 40 °C for 20 min. Again, the glass discs were extensively rinsed with Milli‐Q water. As a last step, the circular glass discs were placed in a mixture of Milli‐Q water, hydrogen peroxide, and ammonia (5:1:1 (v/v/v)) at 80 °C for 20 min, followed by thoroughly rinsing with Milli‐Q water. Finally, the cleaned glass discs were dried under a nitrogen stream. The discs were then stored in a nitrogen‐flushed Petri dish for a maximum of seven days.

Directly before use, the RCA‐cleaned glass discs were again rinsed with Milli‐Q water and ethanol and dried again under a nitrogen stream. Surface activation was achieved by exposure to air plasma (ZEPTO plasma laboratory unit, Diener electronic GmbH & Co. KG, Ebhausen, Germany) for 15 min. For the POx‐HASH particles, the glass discs were prepared as previously described. For the PAAm microgel particles, the plasma‐treated glass discs were additionally functionalized by immersing them in an aqueous solution of PLL‐*g*‐PEG (1 g L^−1^) for at least 30 min. Afterward, the functionalized glass discs were rinsed with Milli‐Q water. For the study of the adhesion force, thin glass cover slips were cleaned and functionalized with PLL‐*g*‐PEG according to the previous description for circular glass discs.

### Inverted Nanoindentation

FluidFM‐based nanoindentation experiments were carried out on a commercial FlexAFM (Nanosurf AG, Liestal, Switzerland) that was mounted on an inverted optical microscope (AxioObserver.Z1, Carl Zeiss AG, Oberkochen, Germany). Both the AFM instrument as well as the microscope were placed on top of a Halcyonics Variobasic (Accurion GmbH, Göttingen, Germany) active vibration insulation. All nanoindentation experiments were performed in a commercially available fluid cell (Asylum Research, Oxford Instruments, Santa Barbara, USA), where the circular glass discs were used as an exchangeable substrate for microgel deposition. Tipless micro‐channeled cantilevers (Micropipettes, Cytosurge AG, Opfikon, Switzerland) with a nominal aperture of 8 µm in diameter and a nominal spring constant of 0.3 N m^−1^ were used. Pressures between +1000 mbar and –800 mbar were applied via a commercially available microfluidic pressure controller (Cytosurge AG, Opfikon, Switzerland).

Before the measurements, the micro‐channeled cantilever was exposed to air plasma for 3 min. Directly afterward, the surface‐activated cantilever was immersed in an aqueous solution of PLL‐*g*‐PEG (1 g L^−1^) for at least 30 min. Then the cantilever was rinsed with Milli‐Q water and mounted on the cantilever holder. The liquid reservoir of the micro‐channeled cantilever was filled with ≈ 30 µL of 1×PBS. The micro‐channel was loaded with 1×PBS by applying an overpressure until the micro‐channel was filled with medium, until a droplet was formed at the aperture.

For the experiments with the POx‐HASH microgel particles, the glass discs were mounted to the fluid cell directly after cleaning and plasma activation, as previously described. For experiments with the PAAm microgel particles, the glass discs were additionally functionalized with PLL‐*g*‐PEG, before mounting to the fluid cell. The fluid cell was then filled with 1×PBS, and 10 µL of the diluted POx‐HASH or PAAm particle suspension was added, respectively. The microgel particles were left to sediment for at least 10 min. The sedimented microgel particles were aspirated to the aperture of the micro‐channeled cantilever by applying an underpressure (−800 mbar) and ejected again by applying an overpressure (+1000 mbar) using the external pressure controller, respectively. The particle manipulation process was followed by optical microscopy. ImageJ was used to determine the particle size from the corresponding optical microscopy images.^[^ ^94^ ^]^ If not stated otherwise, a holding pressure of −100 mbar was applied during the indentation measurements. To study the influence of adhesion between the particle and the substrate on the Young's modulus, the same PAAm microgel particles were not only ramped toward a PLL‐*g*‐PEG‐functionalized glass substrate but also toward a previously plasma‐treated glass substrate without further modification. Therefore, a thin glass cover slip was functionalized with PLL‐*g*‐PEG (preparation as previously described for the circular glass discs) and placed on the plasma‐treated glass disc that was mounted in the fluid cell. Then, the fluid cell was carefully filled with the medium. In this way, the same PAAm microgel particles could be measured against surfaces with various surface modifications. For some experiments, the holding pressure was additionally varied between 0 mbar and −800 mbar in order to investigate its influence on the resulting Young's modulus. The mechanical characterization of the FITC‐PAAm microgel particles was carried out analogously to the approach previously described for PAAm beads.

In order to calibrate the inverted optical lever sensitivity (InvOLS), the tipless micro‐channeled cantilevers were ramped against the hard glass substrate, and the InvOLS was determined from the slope in the region of constant compliance in the force curves. For every microgel particle, the InvOLS was determined before the respective nanoindentation experiment. On three different spots on the substrate, the mean value over 30 consecutive measurements has been determined. The cantilever's spring constant was calibrated according to the method of Cleveland after the nanoindentation experiments.^[^ ^95^ ^]^ Spherical tungsten particles (*n* ≥6) were attached to the free end of the dried cantilever using capillary forces. From the shift of the resonance frequency due to the added mass, the cantilever's spring constant has been determined.^[^ ^95^ ^]^

The data from indentation measurements with a spherical colloidal probe (cf. Figure 3) were taken from a recent paper based on the same batches of microgel particles.^[^ ^35^ ^]^

### Data Analysis

The raw data of photodiode voltage versus z‐piezo displacement were converted to force versus distance curves using home‐written procedures in IGOR Pro (version 8.04, Wavemetrics, Portland, USA). The Young's modulus was determined by fitting the simplified double contact model to the force curves, resulting in force versus indentation curves. The contact point was set by iteratively approximating the data with the chosen contact model. The processed data were fitted to an indentation depth of 5% of the respective particle radius. For both the POx‐HASH and (FITC‐)PAAm microgel particles, the Poisson ratio was set to 0.5, which is a commonly used value for hydrogels.^[^ ^30^, ^96^ ^]^ Regarding the indentation experiments with PAAm on non‐modified glass, the adhesion force was determined from the absolute minimum in the retraction part of the curve, and the work of adhesion from the area between the adhesion peak and non‐contact line, respectively.

### Scanning Electron Microscopy

Scanning electron microscopy images of a micro‐channeled cantilever were acquired with a Leo 1530 VP Gemini (Carl Zeiss AG, Oberkochen, Germany) at an acceleration voltage of 15 kV. The cantilever was sputtered with a platinum layer of ≈1 nm thickness (Sputter Coater 208HR, Cressington Scientific Instruments, Watford, UK).

### Total Internal Reflection Fluorescence (TIRF) Microscopy

WillCo‐dish glass‐bottom dishes (GWST‐3522, WillCo Wells B.V., Amsterdam, Netherlands) were used for investigating the FITC‐PAAm particles with TIRF microscopy. Before use, the dishes were rinsed with ethanol and dried under a nitrogen stream. The glass surfaces were activated by exposing the dishes to air plasma (Zepto, Diener Electronics, Ebhausen, Germany) for 20 min. Additionally, some of the plasma‐treated glass bottom dishes were functionalized with PLL‐*g*‐PEG (1 g L^−1^) by immersing for 30 min. Afterward, the functionalized glass‐bottom dishes were rinsed with Milli‐Q water. Directly after plasma activation or modification with PLL‐*g*‐PEG, respectively, the microgel particle suspension (20 µL) was dropped onto the glass, and 1×PBS (150 µL) was added to prevent drying. The dish was filled with 1×PBS after waiting for 5 min, allowing the particles to sediment.

For imaging the contact area of the FITC‐PAAm particles on plasma‐treated and PLL‐*g*‐PEG‐functionalized glass, a Leica DMi8 Infinity TIRF microscope (Leica Microsystems GmbH, Wetzlar, Germany) equipped with a DFC9000GT‐VSC13730 camera was used. For the oil immersion objective (HC PL APO 100×1.47 Oil, Leica Microsystems GmbH, Wetzlar, Germany), an immersion oil with a refractive index of 1.518 was utilized. The FITC dye molecules in the labeled PAAm particles were excited using a 488 nm laser. The TIRF angle was automatically calibrated before the measurement. Then, TIRF images were acquired, whereby the evanescent wave was set to a penetration depth of 70 nm. The TIRF illumination was optimized for every image by manually adjusting the azimuth. All images were evaluated with ImageJ (version 2.1.0).

## Conflict of Interest

The authors declare no conflict of interest.

## Supporting information



Supporting Information

## Data Availability

The data that support the findings of this study are available from the corresponding author upon reasonable request.
