# Supplementary material for: High‐Throughput Mechanical Characterization of Single Microgel Particles by Fluidic Force Microscopy
Source: Small. 2025 Aug 8;21(38):e05367. doi: 10.1002/smll.202505367 (PMC12462606; doi:10.1002/smll.202505367)
Supplement: Supplementary file 1 — Supporting Information [file SMLL-21-e05367-s001.docx]

Supporting Information

High-Throughput Mechanical Characterization of Single Microgel Particles by Fluidic Force Microscopy

Agnes Specht, Steffen Trippmacher, Nadine Raßmann, Tamino Rößler, Katinka Theis, Krystyna Albrecht, Nicolas Helfricht, Jürgen Groll, and Georg Papastavrou*

**S1 Additional Experimental Methods**

*Materials:* 2-Propanol (p.a., VWR International SAS, Briare France), SU-8 2015 photoresist (micro resist technology GmbH, Berlin, Germany), SU-8 2050 photoresist (micro resist technology GmbH, Berlin, Germany), SU-8 developer (micro resist technology GmbH, Berlin, Germany), Sylgard® 184 elastomer kit (Mavom GmbH, Steinfurt, Germany), and microscope glass slides (ISO 8037/1, thickness of 1 mm, Epredia Netherlands B. V., Breda, Netherlands) were used as received.

*Fabrication of the Microfluidic Device for the PAAm Particle Synthesis:* Single-layer PDMS chips were used to synthesize PAAm microgel particles. For the fabrication of the master mold, a silicon wafer (∅ 4 in, CrysTec GmbH Kristalltechnologie, Berlin, Germany) was rinsed with 2-propanol and dried under a nitrogen stream. A 30 µm thick layer of SU-8 2015 photoresist was applied to the wafer *via* spin coating (1000 rpm, 30 s). The wafer was then soft-baked on a hot plate (95 °C, 4 min). The design of the microfluidic device was exposed to the wafer (12 s, 365 nm wavelength, 18.6 mW cm^2^ intensity) by means of a chrome photomask (JD photo data, Hitchin, UK) and a MJB4 mask aligner (SUSS MicroTec SE, Garching, Germany). Again, the wafer was baked on a hot plate (95 °C, 5 min). While gently agitating, the master structures were developed in SU-8 developer (3 min). After cleaning with 2-propanol and drying under nitrogen, the developed structures were hard-baked on a hot plate (150 °C, 15 min).

A mixture of Sylgard^®^ 184 elastomer base and curing agent (10:1 (w/w)) was poured onto the master mold. The elastomer mixture was then degassed by placing the mold in a desiccator under reduced pressure for 1 h. After curing (150 °C, 1 h), the PDMS chip was removed from the master mold by cutting. A biopsy puncher (Ø 0.75 mm, Micro to Nano BV, Haarlem, Netherlands) was utilized to punch holes into the inlet and outlet chambers in order to be able to connect these to tubings. The PDMS was sonicated in 2-propanol (15 min) and dried in an oven overnight (90 °C).

Microscopy glass slides were used to seal the PDMS micro-channels. Therefore, the PDMS was activated with air plasma (power: Hi, 30 s equilibration time, 30 s plasma time, 380 mTorr pressure, PDC-32G plasma cleaner, Harrick Plasma, Ithaca, NY, USA). The sealed PDMS was dried in an oven overnight (100 °C). The final microfluidic device included two inlet chambers, one outlet chamber, two perpendicular channels (40 µm width) at the cross-junction, and an orifice (10 µm width) which supported the droplet formation.

*Fabrication of the Microfluidic Device for the POx-HASH Particle Synthesis:* Multi-layer PDMS chips were used to synthesize POx-HASH microgel particles. In these multi-layer chips, the aqueous phase flows through a central channel, while the oil phase flows in top and bottom channels, leading to a 3D-focusing at the cross-junction.^[92]^ For the fabrication of the master mold, a silicon wafer (∅ 4 in, CrysTec GmbH Kristalltechnologie, Berlin, Germany) was rinsed with 2-propanol and dried under a nitrogen stream. At first, a 50 µm thick layer of SU-8 2050 photoresist was applied to the wafer *via* spin coating (2500 rpm, 30 s). The wafer was then soft-baked on a hot plate (first: at 65 °C for 6 min, then: at 95 °C for 18 min). The design of the microfluidic device was exposed to the wafer (12 s) by means of a MicroWriter ML3 (Durham Magneto Optics Ltd., UK) that was equipped with a light source (385 nm wavelength, assumption of 3000 mJ cm^-2^ resist sensitivity). The digital photomask had been designed in AutoCAD (Autodesk, Inc., USA). Again, the wafer was baked on a hot plate (first: at 65 °C for 1 min, then: at 95 °C for 5 min). After letting the wafer cool down to room temperature, the second layer of photoresist was added. A 70 µm thick layer of SU-8 2050 photoresist was applied to the wafer *via* spin coating (2500 rpm, 30 s). The wafer was soft-baked, exposed, and post-baked, as previously described. The virtual mask alignment function of the MicroWriter was used to align the second layer on the top layer. While gently agitating, the master structures were developed in SU-8 developer (12 min). The developed structures were rinsed with 2-propanol and dried under nitrogen.

A mixture of Sylgard® 184 elastomer base and curing agent (10:1 (w/w)) was poured onto the master mold. The elastomer mixture was then degassed by placing the mold in a desiccator under reduced pressure for 1 h. After curing (150 °C, 1 h), the PDMS chip halves were removed from the master mold by cutting. A biopsy puncher (Ø 0.5 mm, Micro to Nano BV, Haarlem, Netherlands) was utilized to punch holes into the inlet and outlet chambers in order to be able to connect these to tubings. The PDMS chip halves were sonicated in 2-propanol (15 min) and dried in an oven overnight (90 °C).

For chip bonding, the PDMS chip halves were activated with air plasma (power: Hi, 30 s equilibration time, 30 s plasma time, 380 mTorr pressure, PDC-32G plasma cleaner, Harrick Plasma, Ithaca, NY, USA). Therefore, Milli-Q water (30 µL) was pipetted onto the bottom chip half. Self-locking structures at the sides of each chip were used to align the chip halves under optical control. Finally, the aligned PDMS chips were dried in an oven overnight (100 °C). The final microfluidic device included two oil phase inlet chambers, one aqueous phase inlet chamber, one outlet chamber, and an orifice (60 µm width) at the cross-junction which supported the droplet formation.

**S2 Force *vs.* Indentation Curve and Hertz Exponent for PAAm**

In Figure 2, an exemplary force *vs.* indentation curve of ‘inverted’ nanoindentation with a POx-HASH microgel particle is shown, together with the corresponding Hertz power law coefficient fitted to the logarithmized data. The respective data of ‘inverted’ nanoindentation with a PAAm microgel particle are depicted in the following Figure S2.


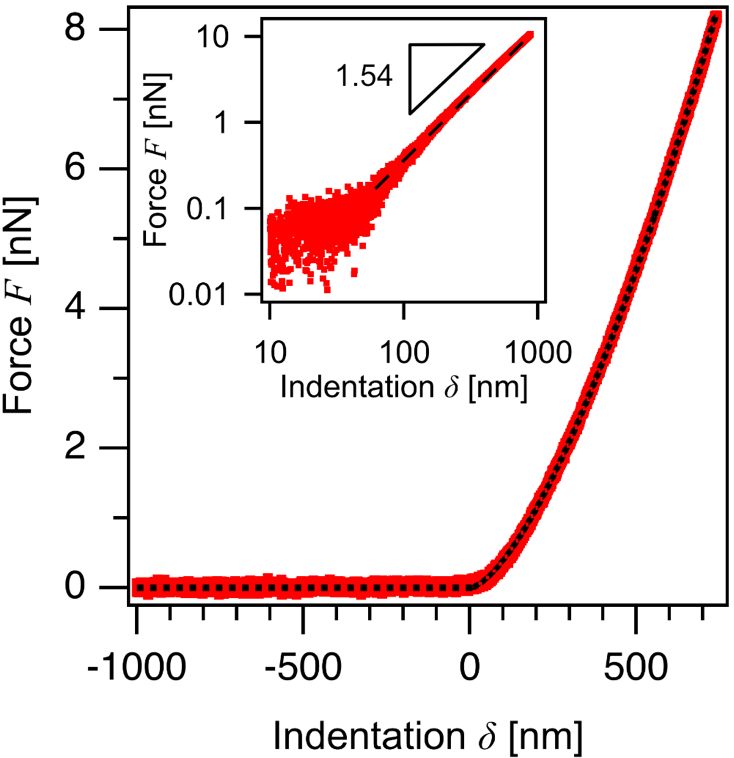


**Figure S2:** Exemplary force *vs.* indentation approach curve measured with a PAAm microgel particle by inverted nanoindentation. The corresponding fit according to the simplified double contact model is illustrated by a dashed line. The inset illustrates the Hertz power law coefficient of 1.54 fitted to the logarithmized data.

**S3 Influence of Adhesion on the Derived Young’s Modulus: Adhesion Study for POx-HASH**

The influence of adhesion on various surfaces, namely PLL-*g*-PEG and bare glass, was studied not only for the PAAm microgel particles (cf. chapter 2.4.) but also for the POx-HASH beads. The corresponding POx-HASH data are shown in Figure S3.


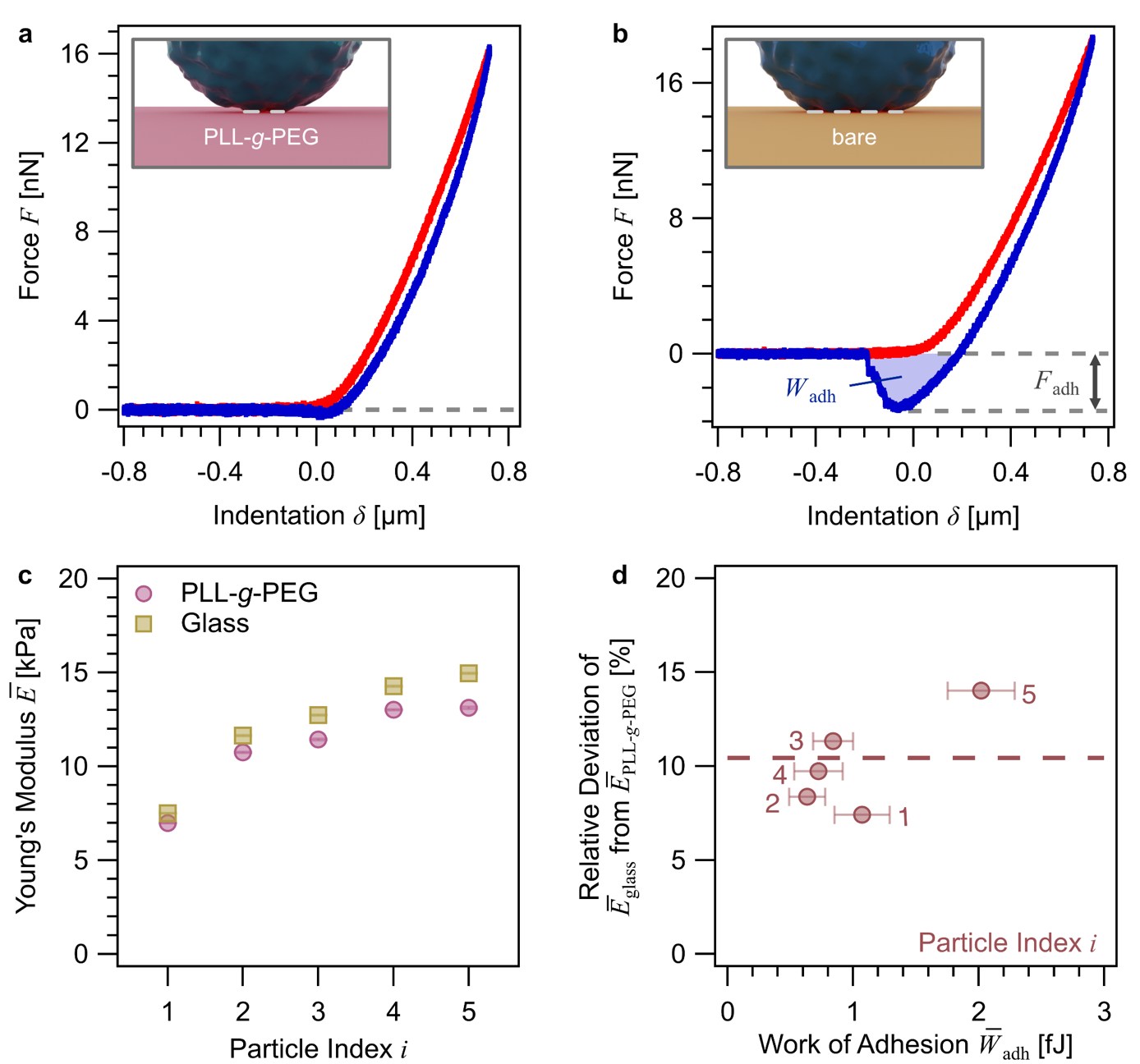


**Figure S3:** Comparison of inverted nanoindentation measurements with POx-HASH particles on different surfaces, namely **a** PLL-*g*-PEG-functionalized and **b** bare glass. The corresponding force *vs.* indentation curves are shown. Schematic representations of the particles’ contact area with the differently modified glass surfaces are depicted as insets. **c** Young’s moduli of five different particles, each measured *vs.* a PLL-*g*-PEG-functionalized and a bare glass surface, respectively. **d** Relative deviation of Young’s modulus measured *vs.* bare glass compared to that *vs.* PLL-*g*-PEG as a function of the respective work of adhesion.

**S4 Influence of Adhesion on the Derived Young’s Modulus: Corresponding Force *vs.* Distance Curves**

In Figure 4a and 4b, force *vs.* indentation curves of PAAm particles measured against PLL-*g*-PEG-functionalized and bare glass are shown, respectively. Figure S2a and S2b depict the corresponding force *vs.* distance curves.


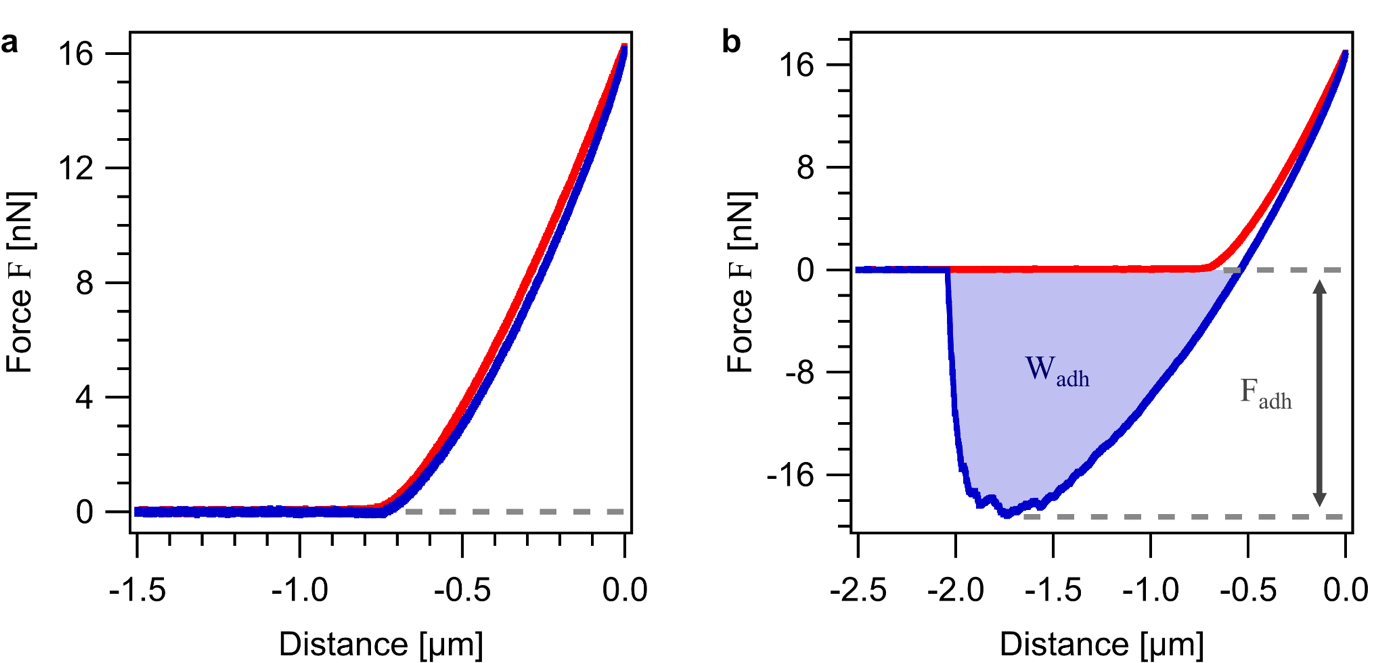


**Figure S4:** Direct force measurements with PAAm microgel particles on different surfaces. Force *vs.* distance curves measured against **a** PLL-*g*-PEG-modified and **b** plasma-treated, bare glass.

**S5 TIRF Data Evaluation**

For the determination of the contact radius from the TIRF images, we evaluated both datasets (PLL-*g*-PEG and bare glass) in the same way. For every particle a section through the center of the particle was drawn in the ImageJ software. A mean filter (3 pixels) was applied, and the intensity profiles were plotted and further analyzed in IGOR Pro (Wavemetrics). Figure S5a and b show the intensity profiles of the two exemplary FITC-PAAm particles depicted in Figure 5a and b, respectively. A threshold of 60 % of the maximum intensity (cf. dashed lines in Figure S5a and b) was chosen for the evaluation of the contact radius *R*_cont_. Asymmetric intensity profiles in the PLL-*g*-PEG data were excluded from further analysis. The particle radius *R* was obtained by evaluation of the transmission light microscopy images (cf. bottom left in Figure 5a and b). The radius ratio *r* = *R*_cont_/*R* is plotted in Figure S5c and d for both surface modifications, respectively.

**
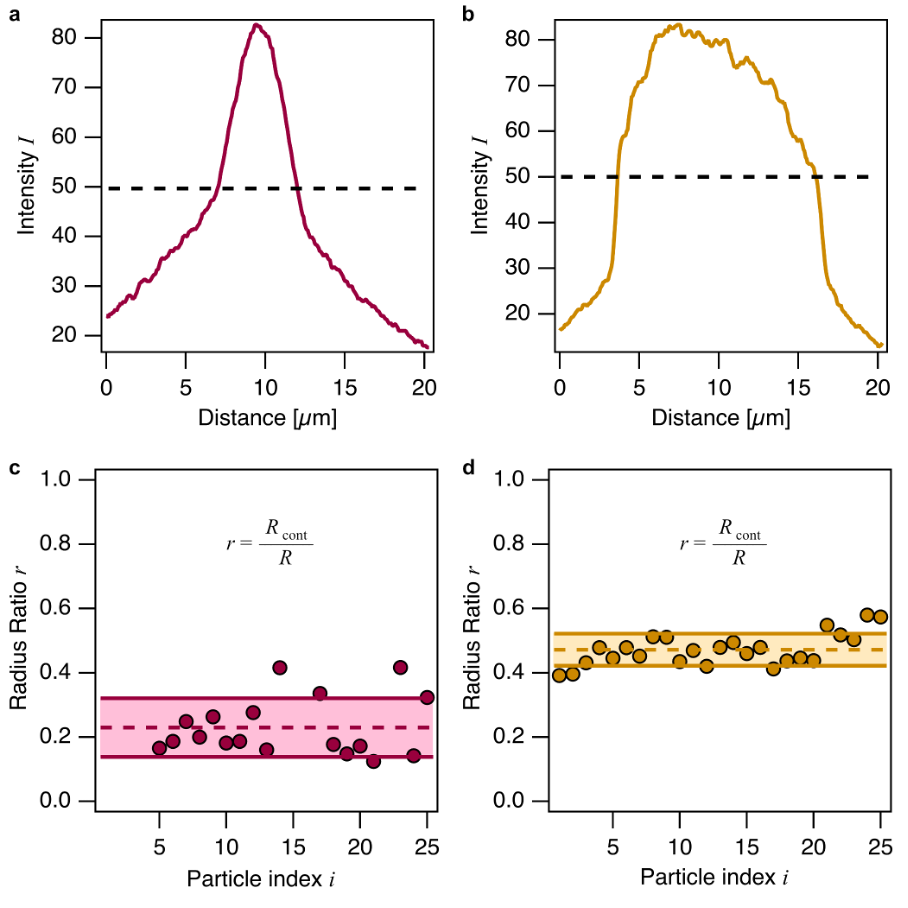
**

**Figure S5:** TIRF-intensity profiles of FITC-PAAm particles on **a** PLL-*g*-PEG modified and **b** bare glass as shown in Figure 5 (bottom right of a and b, respectively). The dashed lines mark the threshold of 60 % of the maximum intensity which was used for the evaluation of the contact radius. Distribution of the radius ratio *r* for FITC-PAAm particles on **c** PLL-*g*-PEG modified and **d** bare glass. The dashed lines represent the average ± one standard deviation (solid lines).

**S6 Influence of the Holding Pressure on the Derived Young’s Modulus: Slopes of the Linear Fits**

In Figure 6, the relative deviation of the Young’s modulus is shown as a function of the applied holding pressure. From the linear fit of the data, we get the following slopes, $m$, for POx-HASH and PAAm, respectively:

POx-HASH: $y=-0.024 x-1.94$

$$m_{POx-HASH}=\left( 0.0237\pm0.0015 \right)\frac{\%}{mbar\left[ negative pressure \right]}$$

PAAm: $y=-0.023 x-2.34$

$$m_{PAAm}=\left( 0.0229\pm0.0006 \right)\frac{\%}{mbar\left[ negative pressure \right]}$$
